# Single-molecule visualization of human A_2A_ adenosine receptor activation by a G protein and constitutively activating mutations

**DOI:** 10.1038/s42003-023-05603-6

**Published:** 2023-11-30

**Authors:** Shushu Wei, Niloofar Gopal Pour, Sriram Tiruvadi-Krishnan, Arka Prabha Ray, Naveen Thakur, Matthew T. Eddy, Rajan Lamichhane

**Affiliations:** 1 Department of Biochemistry & Cellular and Molecular Biology, College of Arts and Sciences, University of Tennessee, Knoxville, TN USA; 2 Department of Chemistry, College of Liberal Arts and Sciences, University of Florida, Gainesville, FL USA

**Keywords:** Single-molecule biophysics, Molecular biophysics

## Abstract

Mutations that constitutively activate G protein-coupled receptors (GPCRs), known as constitutively activating mutations (CAMs), modify cell signaling and interfere with drugs, resulting in diseases with limited treatment options. We utilize fluorescence imaging at the single-molecule level to visualize the dynamic process of CAM-mediated activation of the human A_2A_ adenosine receptor (A_2A_AR) in real time. We observe an active-state population for all CAMs without agonist stimulation. Importantly, activating mutations significantly increase the population of an intermediate state crucial for receptor activation, notably distinct from the addition of a partner G protein. Activation kinetics show that while CAMs increase the frequency of transitions to the intermediate state, mutations altering sodium sensitivity increase transitions away from it. These findings indicate changes in GPCR function caused by mutations may be predicted based on whether they favor or disfavor formation of an intermediate state, providing a framework for designing receptors with altered functions or therapies that target intermediate states.

## Abstract (web summary)

Single-molecule fluorescence identifies how a G protein and constitutively activating mutations impact GPCR conformational equilibria by altering the population of a crucial intermediate state essential for activation.

## Introduction

Inherited and acquired mutations in cell surface receptors often disrupt intracellular signaling, which underlies a wide range of diseases, including developmental growth diseases and cancers. With more than 800 members, human G protein-coupled receptors (GPCRs) are cell surface receptors that participate in numerous and diverse physiological processes and constitute the largest family of druggable proteins^1^. While GPCR signaling is typically activated by agonists, such as small molecules or polypeptides, GPCRs also exhibit basal activity in the absence of external stimulation, the extent of which varies among receptors^2,3^. Mutations can alter basal level signaling, with some mutations leading to reduction or loss of signaling, while other mutations, known as constitutively activating mutations (CAMs), increase receptor basal activity. CAMs can increase basal activity by increasing receptor cell surface expression or promoting receptor transport, or by altering the conformational equilibria between different receptor functional states^4–6^. The mechanisms by which CAMs alter the dynamic equilibrium of GPCR conformers are poorly understood.

CAMs resulting in agonist-independent gain-of-function have been linked to various diseases, such as hyperthyroidism resulting from mutations in the thyrotropin receptor^7^, dwarfism resulting from mutations in the parathyroid hormone receptor^8^, and hypocalcemia resulting from mutations in the calcium-sensing receptor^4^. CAMs are also strongly associated with the onset and progression of numerous cancers, including mutations in α_1B_-adrenergic receptor that promote tumorigenicity^9^ and mutations in the smoothened receptor that contribute to drug-resistant basal cell carcinoma^10^. Additionally, certain human viruses encode for constitutively active receptors, such as herpesvirus KSHV, which produces a constitutively active GPCR associated with Karposi’s sarcoma^11^, and the Epstein-Barr virus, which encodes constitutively active BILF1^12^. While evidence from pharmacological studies across various receptor types highlights the role of constitutively activating mutations in abolishing a receptor’s response to drug efficacy, the mechanisms responsible for this action are not entirely understood.

The structural basis for constitutive activity is so far limited to crystal structures of three distinct GPCRs: rhodopsin^13^, the neurotensin receptor NTSR1^14^, and the A_2A_ adenosine receptor (A_2A_AR)^15^. This is compared to the crystal and cryo-EM structures of more than 100 unique receptors and more than 1000 structures of GPCRs with ligands or partner proteins in total. In this study, we apply single-molecule fluorescence (SMF) to provide additional insight into the structural basis of GPCR constitutive activity.

The human A_2A_AR belongs to the class A, or rhodopsin-like GPCRs, and is a validated target for Parkinson’s disease^16–18^, as well as a target for treating multiple cancers^19–21^. Understanding the mechanisms of A_2A_AR signaling has been the focus of a growing number of spectroscopic approaches, especially nuclear magnetic resonance (NMR) spectroscopy^22–32^. Single-molecule fluorescence (SMF) provides unique and complementary information on the pathways connecting distinct conformational states and the order in which they are populated^33,34^. Total internal reflection SMF, in particular, has enabled the observation of slow conformational changes of the human β_2_-adrenergic receptor that correlated with global structural rearrangements and activation of the receptor^35–37^. Complementing the approach using TIRF, diffusion-based experimental designs have also observed A_2A_AR dynamics on faster, sub-millisecond timescales^38,39^.

Using TIRF SMF, slow, reversible exchange among three fluorescence intensity states was observed for human A_2A_AR bound to agonists within lipid nanodiscs^34^. This exchange revealed that the population of an active-like A_2A_AR conformation proceeded through an intermediate state, with direct transitions between inactive and active-like conformations being rare^34^. These findings established a conceptual framework for comprehending A_2A_AR activation as a step-wise, reversible process. In this process, small molecule agonists enhanced the frequency of transitions from an inactive state to an intermediate state before ultimately reaching an active conformation. We leveraged this framework to investigate whether CAMs altered this step-wise process or circumvented it altogether. Additionally, we applied the same framework to distinguish the effects of CAMs from the influence of ternary complex formation with a G protein on the A_2A_AR activation mechanism.

These observations were compared with single-molecule fluorescence measurements of an A_2A_AR variant that uncouples ligand-binding activity from signaling. Notably, this variant exhibited meaningful differences in the rate constants between the inactive state and intermediate state compared to A_2A_AR variants containing CAMs. Through quantitative analysis of state lifetimes and their exchange rates, we uncovered that both mutations and the addition of a G protein substantially impact the conformational equilibria between the inactive and intermediate states, while only marginally affecting the equilibria between the intermediate and active states. Our data yield valuable mechanistic insights into the sequence of events required for GPCR activation and offer potential insights into pharmacological intervention in CAM-associated diseases.

## Results

### Single-molecule fluorescence with constitutively active A_2A_AR variants in lipid nanodiscs

**Fig. 1:**
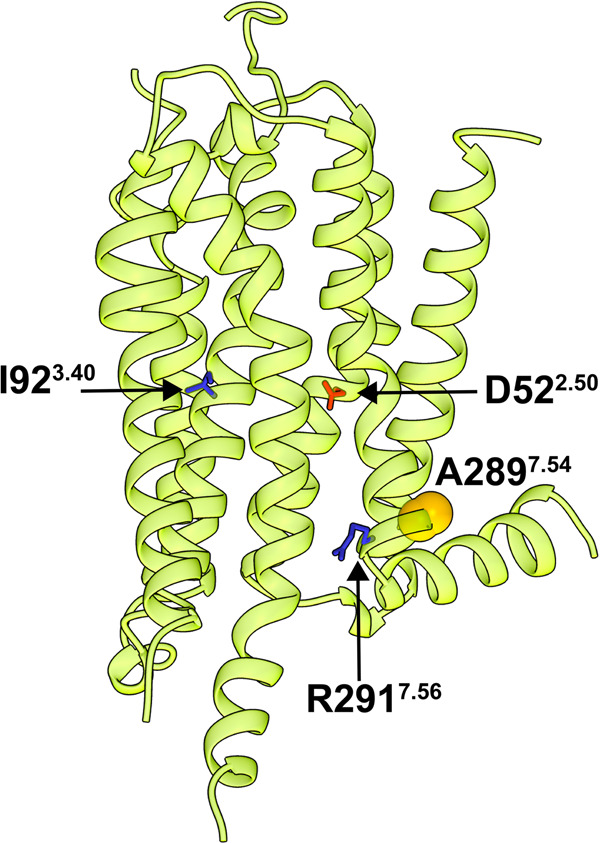
Locations of A289 and residues modified to study signaling mechanisms in a crystal structure of human A_2A_AR. A_24_AR in complex with the antagonist ZM241385 is shown in ribbon representation (PDB 3EML). A289 in helix VII is shown in space-filling representation and residues D52, I92 and R291 are shown in stick representation.

For single-molecule fluorescence imaging experiments, we utilized a variant of A_2A_AR that contained a single solvent-accessible cysteine replacement at position 289, located at the intracellular surface of Helix 7 (Fig. 1)^40^, which we refer to as A_2A_AR in the following text. Previous single-molecule fluorescence^34^ and NMR studies^26,29^ demonstrated that structural probes at this location are sensitive reporters to changes in the conformation of A_2A_AR associated with different efficacies of bound ligands. To investigate the impact of activating mutations, we created two additional variants from the A_2A_AR sequence, I92^3.40^N and R291^7.56^Q (superscripts denote the Ballesteros–Weinstein nomenclature for GPCR residue positions). The amino acid replacement I92^3.40^N, located in transmembrane (TM) 3 near the conserved “toggle switch” tryptophan W246^6.48^, was previously shown to increase A_2A_AR basal activity^41^. The second amino acid replacement R291^7.56^Q, located near the intracellular surface of Helix VII, was shown to increase basal activity by disrupting interactions with the adjacent Helix VI and associated lipid molecules^26^. These two variants were selected because they both resulted in increased basal activity, but the locations of the mutations were in very different regions of the receptor (Fig. 1). To provide a point of comparison, we also studied an A_2A_AR variant containing a single inactivating mutation, D52^2.50^N, located in the TM region of Helix II. This variant has been documented to retain native-like ligand binding affinities for both antagonists and agonists but demonstrates a nearly complete loss of G protein signaling activity^30,42^.

Purified A_2A_AR and A_2A_AR variants were reconstituted into lipid nanodiscs containing a 65:30:5 molar ratio mixture of 1-palmitoyl-2-oleoyl-glycero-3-phosphocholine (POPC), 1-palmitoyl-2-oleoyl-sn-glycero-3-phospho-L-serine (POPS), and biotinylated 1-palmitoyl-2-oleoyl-glycero-3-phosphoethanolamine (POPE) lipids, respectively, and shown to produce homogeneous samples (Supplementary Fig. 1). The variant A_2A_AR[R291Q] had been previously shown to retain pharmacological activity highly similar to native A_2A_AR in lipid nanodiscs^26^. Using competitive radioligand experiments, we verified the ligand binding activity of A_2A_AR[I92N] and A_2A_AR[D52N] in the nanodiscs with the same lipid composition as single-molecule fluorescence experiments and found both variants retained native-like pharmacological activity (Supplementary Fig. 2). A_2A_AR and A_2A_AR variants were labeled with the Cy3 fluorophore at position 289, according to previously reported protocols^34,43^. As the Cy3 fluorescence emission intensity depends on the local environment of the fluorophore, changes in the fluorescence emission intensity are strongly correlated with changes in protein structure and the environment around Cy3^36,44,45^, which we observed previously for A_2A_AR labeled at position C289^34^. Cy3-labeled A_2A_AR variants were immobilized on a microscope slide and monitored under TIRF illumination, following established protocols^34,36^. The resolution of the camera used in TIRF imaging experiments facilitated observations of changes in fluorescence emission occurring on the timescale of 100 ms. This capability enabled us to capture processes that occurred on a timescale of 10 s^−1^ or slower, which correlated with function-related conformational rearrangements of the receptor backbone^34,46^. Control experiments were performed to characterize the specific immobilization of biotin-labeled nanodiscs to a PEG-coated quartz slide surface using biotin-streptavidin interactions^34^.

### Constitutively active A_2A_AR variants exhibit transitions among three fluorescence intensity states

**Fig. 2:**
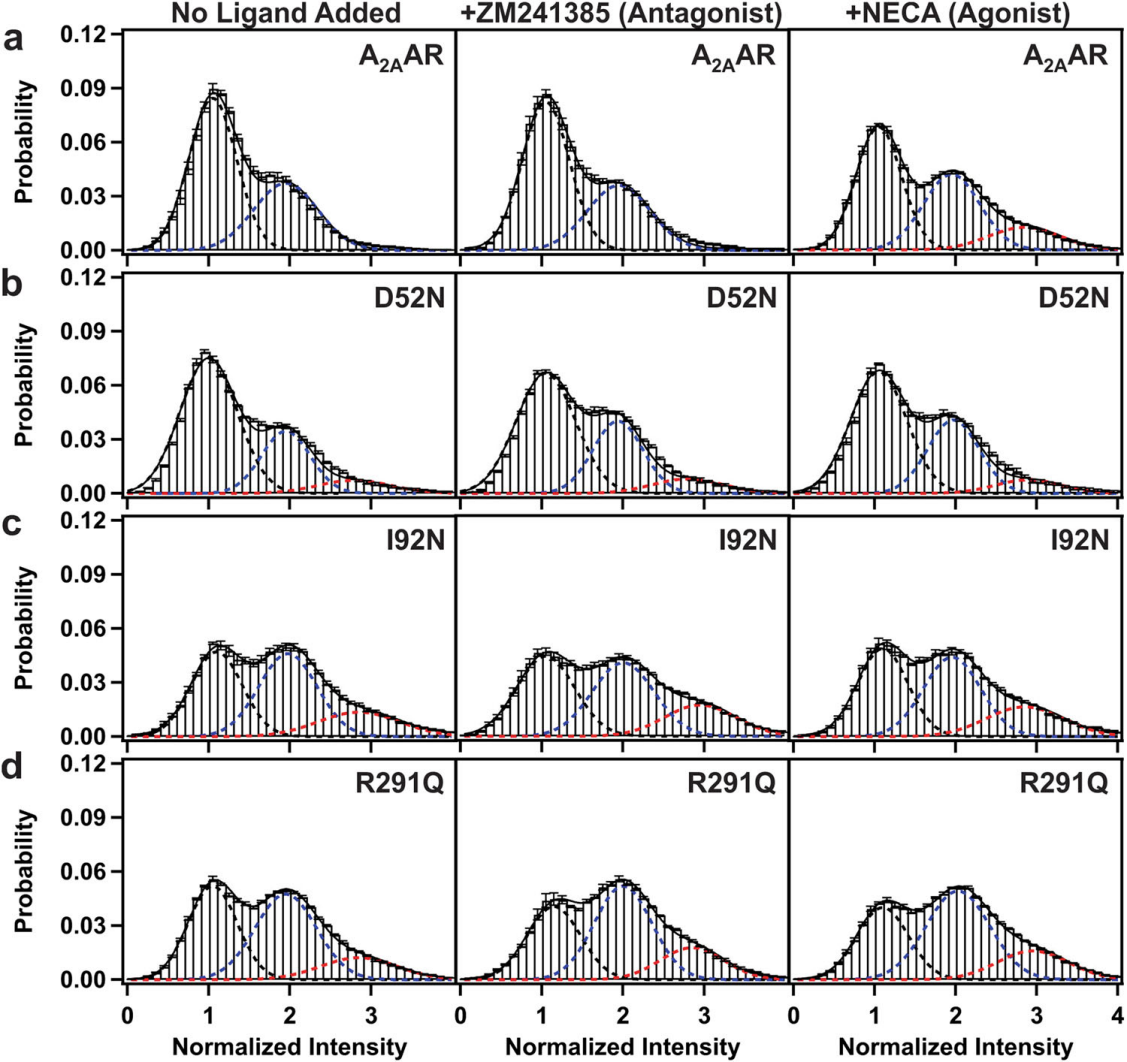
Single-molecule fluorescence histograms compiled from collections of individual molecules of A_2A_AR, A_2A_AR[D52N], A_2A_AR[I92N] and A_2A_AR[R291Q]. **a** Histograms compiled for A_2A_AR with no ligand added (apo), in complex with the antagonist ZM241385, and in complex with the agonist NECA. The dashed lines represent fits to Gaussian populations centered around the normalized intensities of 1 (black), 2 (blue), and 3 (red). The composite fit representing the sum of the Gaussian fitted curves is shown as a solid black line. Error bars represent the standard error calculated for each bar of the histogram. **b**–**d** Histograms compiled for **b** A_2A_AR[D52N], **c** A_2A_AR[I92N] and **d** A_2A_AR[R291Q]. Same presentation details as in (**a**).

Fluorescence emissions were measured, and fluorescence time trajectories were generated for Cy3-labeled A_2A_AR, A_2A_AR[I92N], A_2A_AR[R291Q], and A_2A_AR[D52N] with no ligand added during the sample preparation, i.e., apo preparation, and for complexes with the antagonist ZM241385 and the agonist NECA, which were compared with single-molecule traces recorded with A_2A_AR (Supplementary Fig. 3 and Fig. 2). ZM241385 and NECA were chosen because they have been extensively utilized within the scientific literature for investigating A_2A_AR in pharmacological and biophysical studies and because each share its pharmacophores with the majority of other antagonists or full agonists, respectively^47^.

Following protocols validated in earlier TIRF studies with GPCRs^34,36^, the subsequent analysis focused on data from dynamic molecules from folded and functional receptors. Approximately 40–50% of the trajectories exhibited dynamic behavior while the remaining 50–60% of the trajectories showed a static single-step bleaching behavior without any transitions, consistent with earlier SMF studies of A_2A_AR^34^ and β_2_AR^35,36^.

Previously, two distinct fluorescence emission intensities were observed for A_2A_AR without ligand added (apo) or in complex with the antagonist ZM241385^34^. For agonist complexes with A_2A_AR, a third fluorescence intensity state was also observed, which was assigned to an active or active-like conformation^34^. In contrast to the observations with A_2A_AR, for both variants A_2A_AR[I92N] and A_2A_AR[R291Q], we observed three distinct fluorescence emission intensities not only for agonist-bound preparations but also for complexes with the antagonist ZM241385 and in absence of any ligand added (Supplementary Fig. 3). For A_2A_AR[D52N], we also observed three fluorescence emission intensities for the apo sample and for complexes with the antagonist ZM241385 and agonist NECA (Supplementary Fig. 3).

### Constitutively activating mutations increase the population of an intermediate state required for A_2A_AR activation

Single-molecule fluorescence traces of A_2A_AR, A_2A_AR[D52N], A_2A_AR[I92N], and A_2A_AR[R291Q] were compiled into histograms to facilitate comparison of the relative populations of the observed fluorescence states (Fig. 2, Supplementary Table 3). In the absence of any ligand added (apo), A_2A_AR exhibited two distinct fluorescence emission states centered around two peaks, with relative areas of 63% and 37% for states 1 and 2, respectively. Similarly, for the complex with the antagonist ZM241385, two peaks with relative areas of 63% and 37% for states 1 and 2, respectively, were observed (Fig. 2a and Supplementary Table 2). For agonist-bound A_2A_AR, distinct emission states were observed centered around three peaks, with relative areas of 49%, 37%, and 14% for states 1, 2, and 3, respectively (Fig. 2a and Supplementary Table 2), consistent with previous findings^34^.

Compared with A_2A_AR, the variant A_2A_AR[D52N] exhibited three fluorescence emission intensities not only for the agonist-bound receptor but also for the apo receptor and the complex with the antagonist ZM241385 (Fig. 2b and Supplementary Table 2). Compared to A_2A_AR, for all samples of A_2A_AR[D52N], the peak corresponding to state 1 was increased and the peak corresponding to state 2 exhibited a diminished area (Supplementary Table 2). Furthermore, the peak intensities observed for A_2A_AR[D52N] were highly similar for complexes with antagonists and agonists, indicating that the mutation eliminated the sensitivity of the receptor to the efficacy of bound ligands (Fig. 2b and Supplementary Table 2).

Both variants A_2A_AR[I92N] and A_2A_AR[R291Q] exhibited three fluorescence emission intensities for the apo receptor and for complexes with the antagonist ZM241385 and agonist NECA (Fig. 2c, d and Supplementary Table 2). Notably, for all A_2A_AR variants, including those containing CAMs, no new fluorescence emission states were observed compared with A_2A_AR. Instead, compared to A_2A_AR, for variants containing CAMs, we observed a decrease in the population of state 1, an increase in the population of state 2, and a significant increase in the population of state 3 for apo receptors and for complexes with the antagonist and with the agonist (Supplementary Table 2). Additionally, for both A_2A_AR[I92N] and A_2A_AR[R291Q], we observed similar histograms for apo samples and for complexes with antagonists and agonists, indicating the efficacy of bound ligands did not impact on the relative areas of the three fluorescence emission states.

### A_2A_AR variants containing constitutively activating mutations exhibit reversible fluctuations among three intensity states in sequential order

**Fig. 3:**
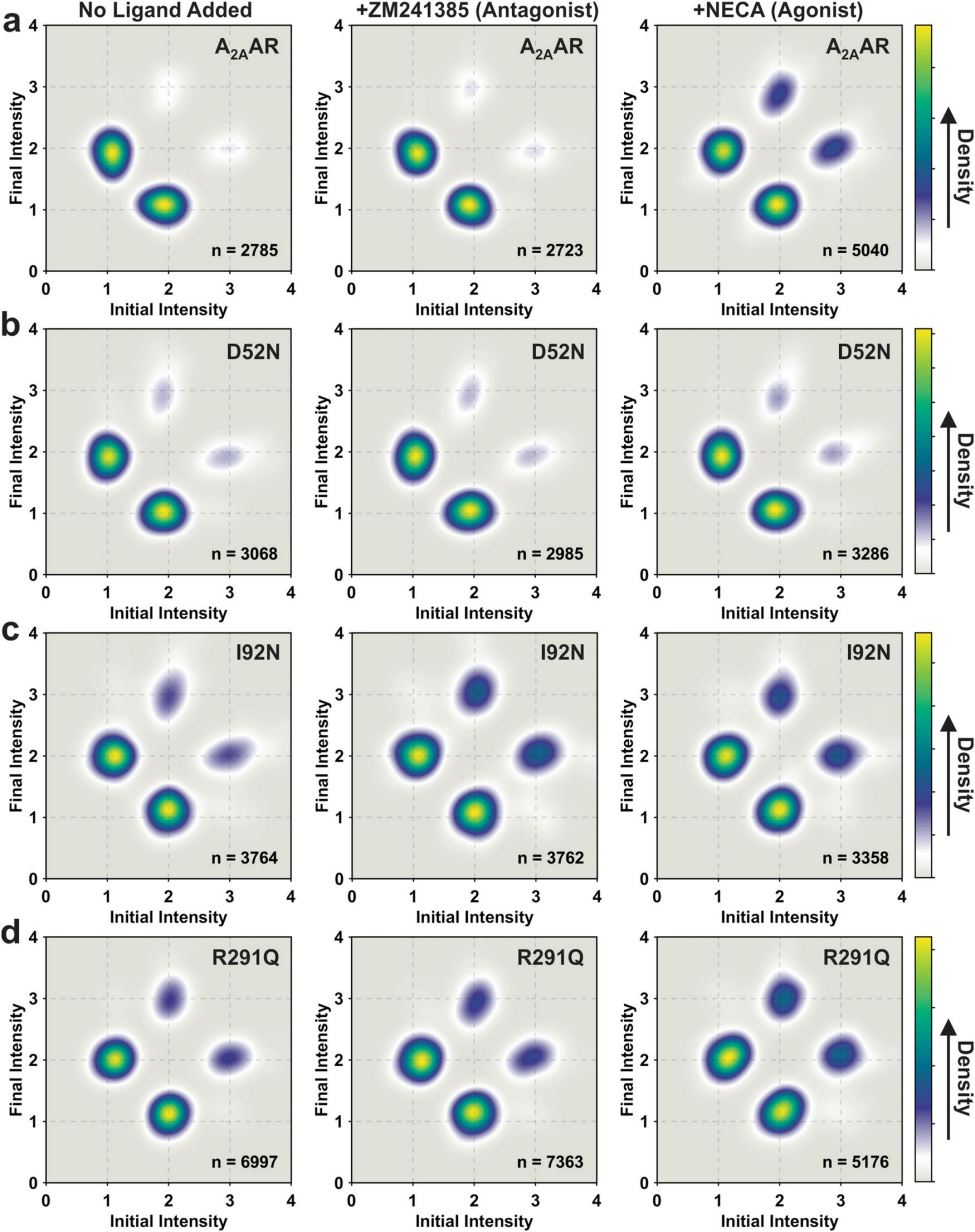
TDPs of A_2A_AR, A_2A_AR[D52N], A_2A_AR[I92N], and A_2A_AR[R291Q] with different ligands or without ligand. TDPs were generated from the normalized initial and final intensities. **a** The TDPs of A_2A_AR without ligand added (apo), in complex with the antagonist ZM241385 and in complex with the agonist NECA. The *X* and *Y* axes of TDP plots represent the normalized initial and final intensities of transition events. The gradient color scheme is indicated in the color bars, where the arrowhead points toward a relatively higher density, which relates to the frequency of observed transitions. “*n*” refers to the number of analyzed transitions. **b**–**d** TDPs for **b** A_2A_AR[D52N], **c** A_2A_AR[I92N], and **d** A_2A_AR[R291Q]. Same presentation details as in (**a**).

Fluorescence intensities for normalized single-molecule time trajectories were fit using a hidden Markov model^48^ (Supplementary Fig. 3). To visualize the transitions between states and the connections among the states, we generated two-dimensional transition density plots (TDPs) using transitions from the fitted traces for A_2A_AR and all A_2A_AR variants (Fig. 3). For A_2A_AR, the TDP for the complex with the agonist NECA was significantly different from the TDP of apo and antagonist-bound A_2A_AR (Fig. 3a), consistent with earlier results^34^. Specifically, agonist-bound A_2A_AR exhibited more frequent transitions to state 3 and spent a longer time in state 3 before transitioning to state 2. Importantly, transitions between states 1 and 3 were infrequent, consisting of fewer than 1% of the total transitions, and thus were not visible in the TDP (Fig. 3a and Supplementary Table 4). Comparatively, for A_2A_AR[D52N], all TDPs for the apo receptor and complexes with the antagonist ZM241385 and agonist NECA appeared highly similar, with only subtle differences observed for the complex with the agonist NECA (Fig. 3b). Transitions between states 1 and 3 were again very rare, consisting of fewer than 2% of the total transitions, analogous to A_2A_AR (Supplementary Table 4).

TDPs for the CAM-containing variants A_2A_AR[I92N] and A_2A_AR[R291Q] appeared similar to each other and also resembled the TDP for agonist-bound A_2A_AR (Fig. 3c and d). Interestingly, unlike A_2A_AR, the TDPs for all samples of A_2A_AR[I92N] and A_2A_AR[R291Q] showed only minor differences, indicating that the activity level of the receptors was not responsive to the efficacy of bound ligands. Notably, transitions between states 1 and 3 were found to be more frequent than for A_2A_AR but still relatively rare, consisting of fewer than 3% of the total transitions (Supplementary Table 4). These results indicate that constitutively activating mutations increase the basal level of receptor signaling by increasing the frequency of transitions to state 2, the conformational intermediate, rather than bypassing transitions to state 2 by increasing the frequency of transitions between states 1 and 3. Therefore, a conceptual framework that describes reversible sequential transitions developed to explain ligand efficacy also appears consistent with the impact of constitutively activating mutations on function-related dynamics.

### The influence of ternary complex formation with an engineered G protein on the fluctuations of A_2A_AR and A_2A_AR variants

**Fig. 4:**
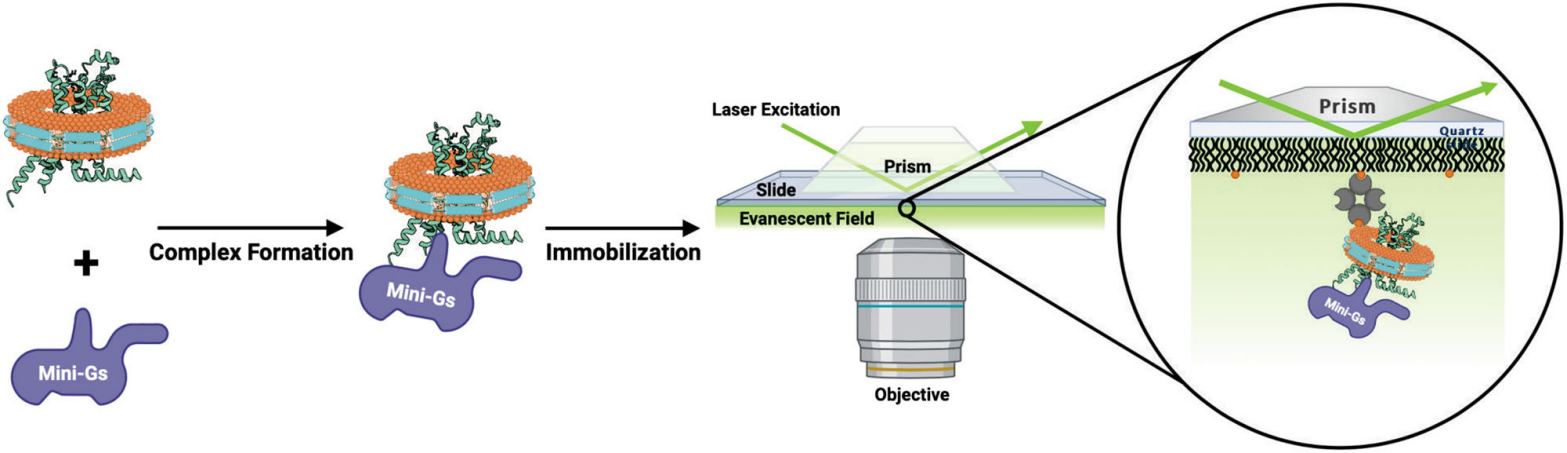
Schematic of SMF experiments with A_2A_AR in a ternary complex with an engineered G protein (mini-G_S_). The receptor in lipid nanodiscs was incubated with mini-G_S_ prior to applying the sample to a channel in a quartz slide for TIRF imaging experiments. This image was prepared with BioRender.

We next applied single-molecule fluorescence to investigate how ternary complex formation with a G protein influenced the conformational exchange of A_2A_AR and the studied A_2A_AR variants. Ternary complexes were formed by incubating the receptor in lipid nanodiscs with “mini-G_S_”, an engineered G protein designed to mimic the binding of full-length Gα_S_ to the receptor^49^. After incubating the proteins together, the resulting complex was then added to the quartz slides for TIRF imaging experiments (Fig. 4). See the “Methods” section for further details.

**Fig. 5:**
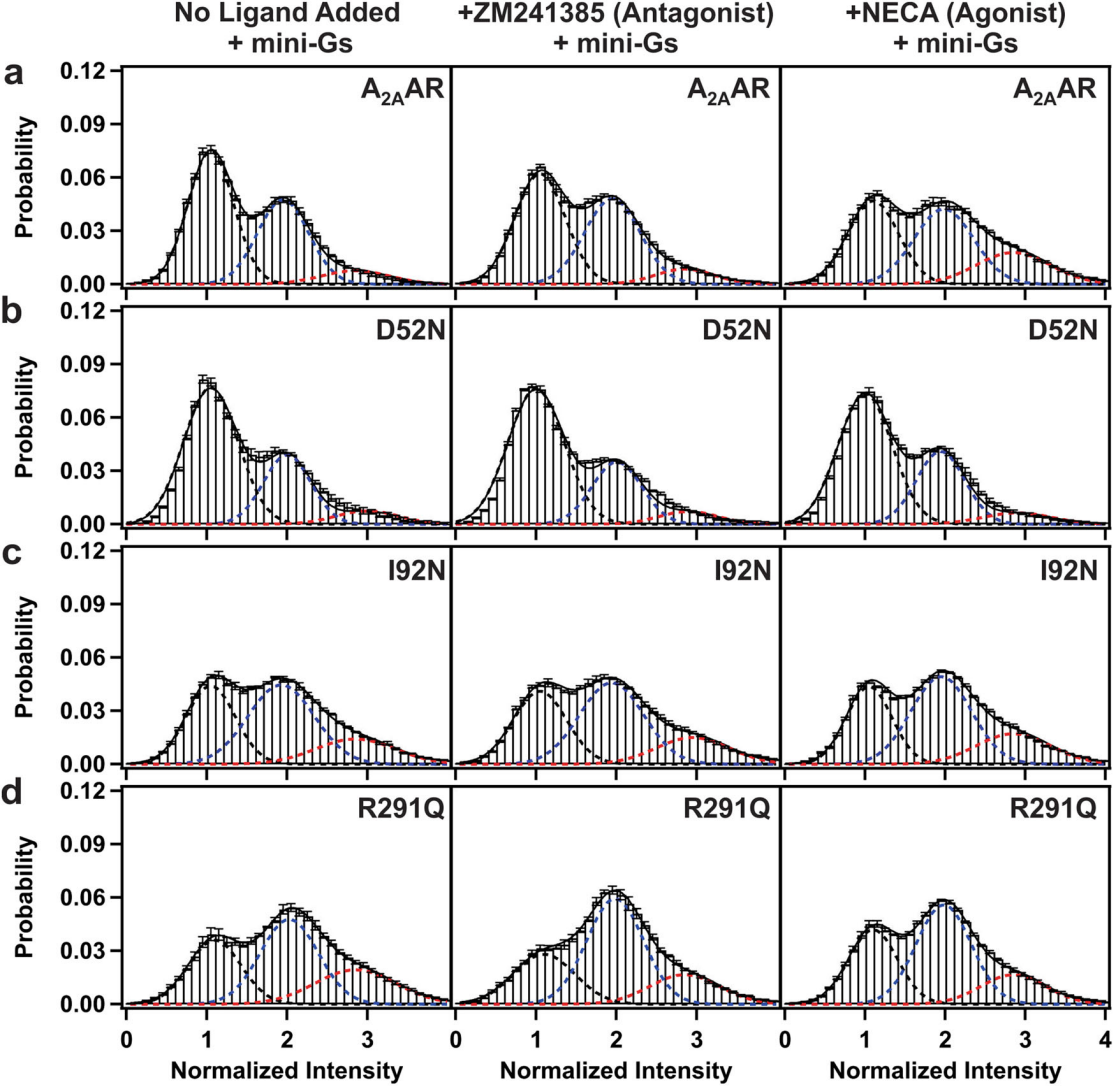
Single-molecule fluorescence histograms compiled from collections of individual molecules of A_2A_AR, A_2A_AR[D52N], A_2A_AR[I92N], and A_2A_AR[R291Q] in the presence of Mini-G_S_. **a** Histograms compiled for A_2A_AR with no ligand added (apo), in complex with the antagonist ZM241385, and in complex with the agonist NECA in the presence of mini-G_S_. Same presentation details as in Fig. 2. **b**–**d** Histograms compiled for **b** A_2A_AR[D52N], **c** A_2A_AR[I92N] and **d** A_2A_AR[R291Q].

The addition of mini-G_S_ to A_2A_AR showed a marked and measurable impact on the relative areas for all fluorescence emission intensities (Fig. 5a). Notably, for all samples of A_2A_AR, the addition of mini-G_S_ increased the area of state 3: for apo and antagonist-bound A_2A_AR, from <1% in the absence of mini-G_S_ to 8% in the presence of mini-G_S_, and for agonist-bound A_2A_AR, from 14% in the absence of mini-G_S_ to 21% in the presence of mini-G_S_ (Fig. 5a and Supplementary Table 2). For all samples of A_2A_AR, in the presence of mini-G_S,_ we also observed a decrease in the area of state 1 and at most, a marginal impact on the area of state 2 (Supplementary Table 2). Notably, the response to the addition of mini-G_S_ to A_2A_AR was distinct from the measured impact of introducing constitutively activating mutations, where we observed an increase in the area of state 2 (Fig. 2).

The response of A_2A_AR to the presence of mini-G_S_ contrasted with that of A_2A_AR[D52N]. For A_2A_AR[D52N], the addition of mini-G_S_ had no measurable impact on the relative populations of the three observed states (Fig. 5b and Supplementary Table 2). This was expected because this variant has been noted to have very low G protein signaling levels even in the presence of a large concentration of a full agonist^42^, likely due to the inability of this variant to form ternary complexes. The impact of this mutation on A_2A_AR has been well-documented^50,51^, and analogous mutations in the same positions of many other class A have also resulted in similar significant alterations to signaling^52^. The precise mechanism of the loss of signaling activity for A_2A_AR[D52N] remains a subject of ongoing investigation, but earlier determination of the crystal structure of A_2A_AR[D52N] in complex with a full agonist pointed to subtle changes in the backbone trajectory around the conserved NPXXY motif^42^, and related NMR experiments noted that this mutation altered function-related conformational dynamics at the receptor intracellular surface^30^.

For both A_2A_AR[I92N] and A_2A_AR[R291Q], the addition of mini-G_S_ also appeared to have a smaller impact on the areas of the three observed fluorescence emission states (Fig. 5c, d and Supplementary Table 2). For A_2A_AR[I92N], a modest increase in the relative area of state 2 and a concurrent decrease in the relative area of state 1 were observed in the presence of mini-G_S_ with no change in state 3 (Fig. 5c). For A_2A_AR[R291Q], we also observed a decrease in the area of state 1 for apo and antagonist-bound A_2A_AR[R291Q] and an increase in the area of state 2 for antagonist-bound and agonist-bound A_2A_AR[R291Q] (Fig. 5d). These data indicate that for the constitutively active A_2A_AR variants, the addition of mini-G_S_ does not further drive the conformational equilibrium.

### Reversible, sequential fluctuations among A_2A_AR conformational states are maintained in the presence of G protein

**Fig. 6:**
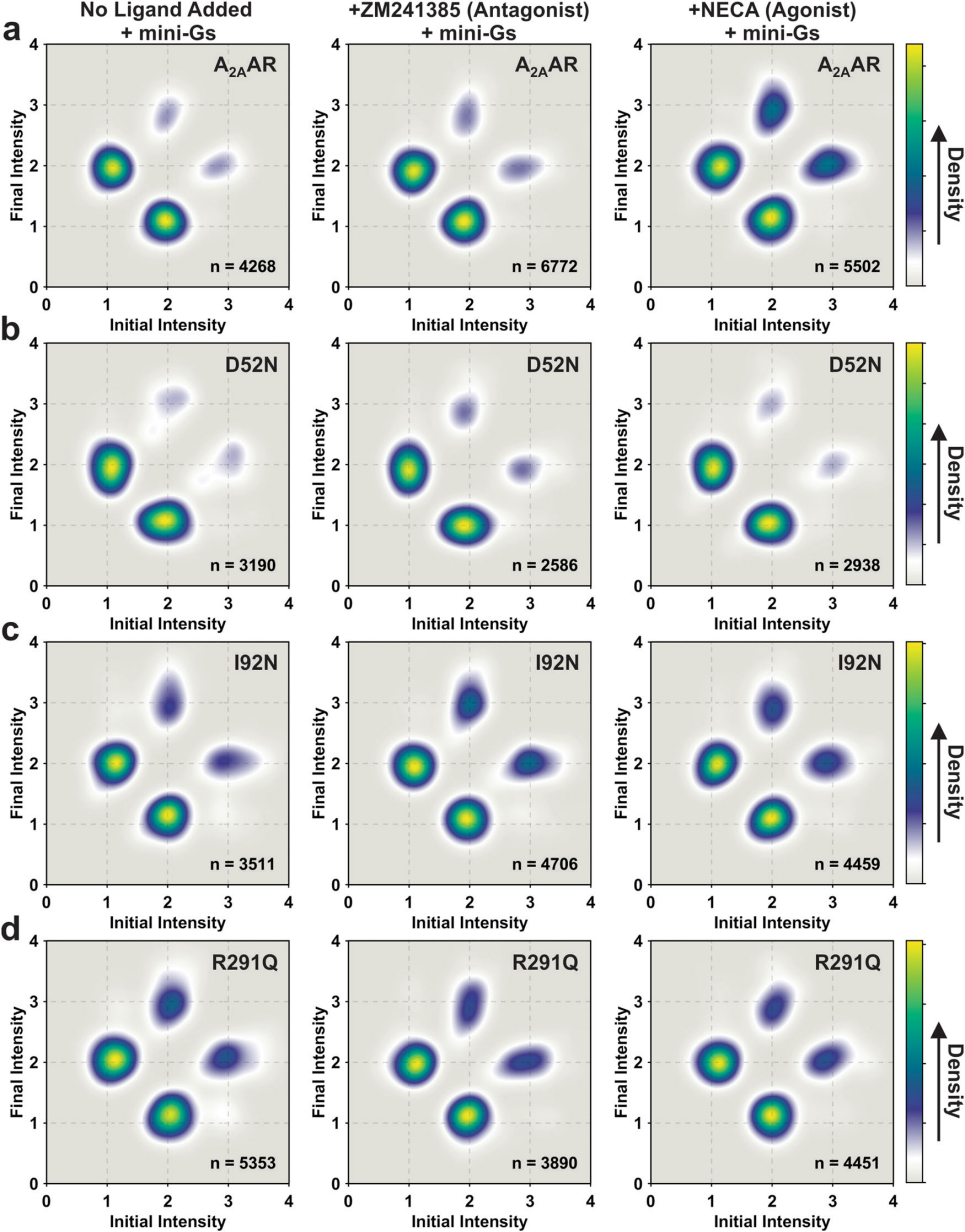
TDPs of A_2A_AR, A_2A_AR[D52N], A_2A_AR[I92N], and A_2A_AR[R291Q] in the presence of mini-G_S_. TDPs were generated from the normalized initial and final intensities. **a** The TDPs of A_2A_AR without ligand added (apo), in complex with the antagonist ZM241385 and in complex with the agonist NECA in the presence of mini-G_S_. The *X* and *Y* axes of the TDP plots represent the normalized initial and final intensities of transition events. The gradient color scheme is indicated in the color bars, where the arrowhead points toward a relatively higher density, which relates to the frequency of observed transitions. “*n*” refers to the number of analyzed transitions. **b**–**d** TDPs for **b** A_2A_AR[D52N], **c** A_2A_AR[I92N], and **d** A_2A_AR[R291Q] in the presence of mini-G_S_. Same presentation details as in (a).

We used the hidden Markov model to fit the normalized single-molecule time trajectories for A_2A_AR samples observed in the presence of mini-G_S_ and generated the corresponding TDPs (Fig. 6). For A_2A_AR in the presence of mini-G_S_, we observed an increased frequency of transitions between states 2 and 3 for both the apo receptor and antagonist-bound A_2A_AR (Fig. 6a). Importantly, transitions between states 1 and 3 were again found to be rare and not populated in the TDP. This indicates that the vast majority of A_2A_AR conformational fluctuations proceeded in a reversible, sequential manner even in the presence of a partner protein.

For A_2A_AR[D52N], A_2A_AR[I92N], and A_2A_AR[R291Q], we observed at most only minor differences in the TDPs recorded for samples prepared in the absence and presence of mini-G_S_ (Fig. 6b–d). As with A_2A_AR, there was no measurable increase in the frequency of transitions between states 1 and 3 for all variants in the presence of mini-G_S_, consistent with reversible sequential transitions between states.

### Constitutively activating mutations and the G protein shift the equilibrium towards the intermediate state

From statistical analysis of the dwell times of collections of receptors, we determined the average time spent or mean occupancy time in each state and the kinetics of transitioning among states 1–3 for A_2A_AR and all A_2A_AR variants (Supplementary Fig. 6 and Supplementary Table 5). For A_2A_AR, the average time spent in state 1 was longer (~2.9 s) compared to the average time spent in states 2 or 3 for the apo receptor and for both antagonist-bound A_2A_AR and agonist-bound A_2A_AR (Supplementary Fig. 6a and Supplementary Table 5). Similarly, the A_2A_AR[D52N] variant also spent a longer time in state 1 (~2.9 s) (Supplementary Fig. 6a and Supplementary Table 5). Interestingly, for both the A_2A_AR[I92N] and A_2A_AR[R291Q] variants, we observed a shorter mean occupancy time (~1.5 s) compared with both A_2A_AR and A_2A_AR[D52N] (Supplementary Fig. 6a and Supplementary Table 5). While the addition of mini-G_S_ appeared to shorten the mean occupancy times for A_2A_AR, especially for agonist-bound A_2A_AR (~1.8 s), there was no impact of the addition of mini-G_S_ for A_2A_AR[D52N], A_2A_AR[I92N] or A_2A_AR[R291Q] (Supplementary Fig. 6b and Supplementary Table 5).

For nearly all samples of A_2A_AR and A_2A_AR variants, we observed that the dwell time histograms of states 1 and 2 do not fit well with a mono-exponential function. Instead, a reduced chi-squared analysis showed that a bi-exponential function consisting of faster and slower transitions provided the best fit (Supplementary Tables 7–14). Three exceptions were observed where a mono-exponential function appeared to provide a better fit for transitions state 2 to state 1: A_2A_AR in complex with the antagonist ZM241385, A_2A_AR[I92N] in complex with the agonist NECA and A_2A_AR[I92N] in complex with NECA in the presence of mini-G_S_ (Supplementary Tables 7–14). Since both shorter and longer dwell times were observed within the same molecule, most likely this indicated that states 1 and 2 each contain two kinetically distinct substates that share the same fluorescence intensity.

**Fig. 7:**
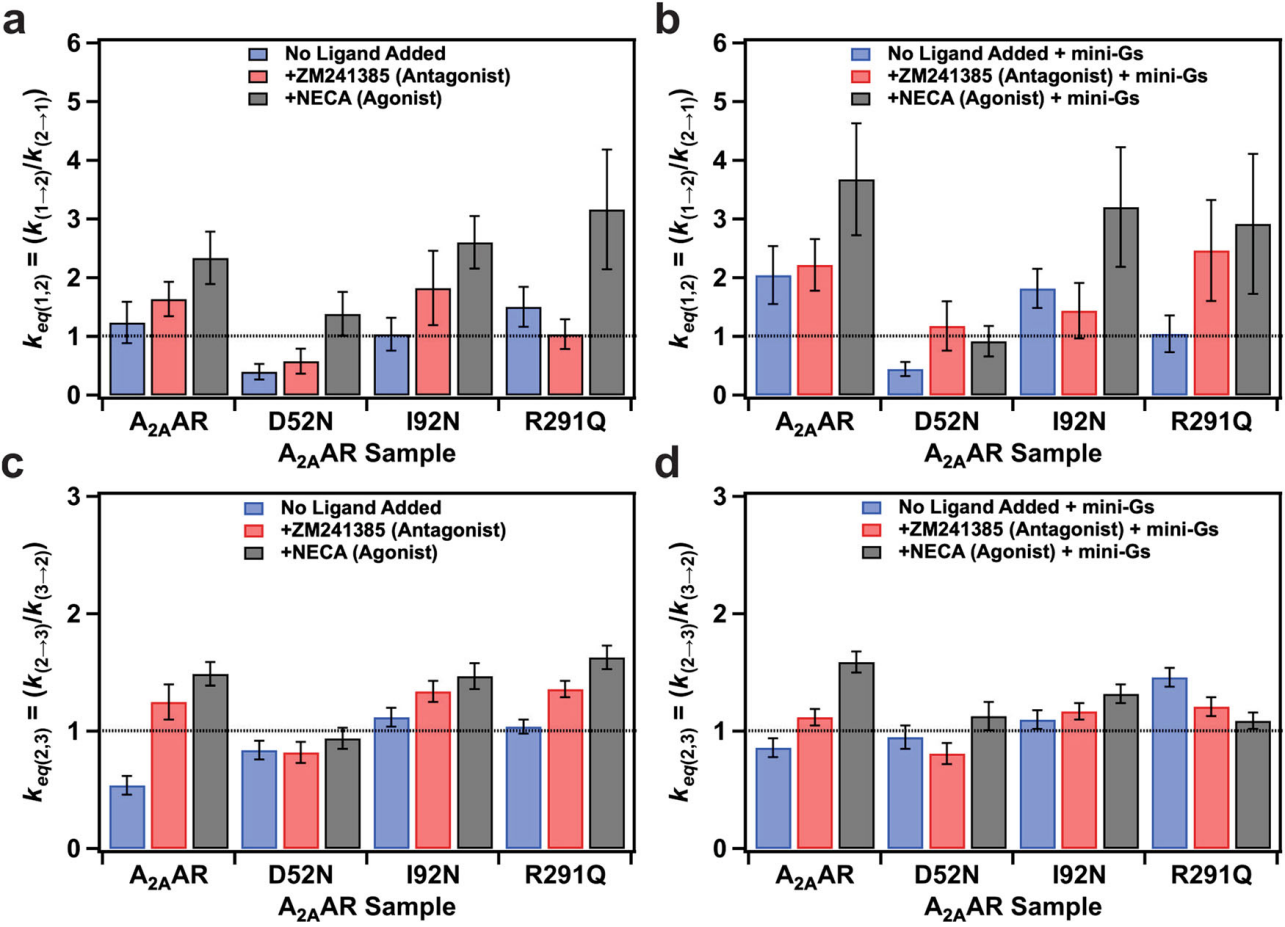
Ratios of equilibrium rate constants between consecutive states. **a** and **b** Ratio of transition rate constants between states 1 and 2 for samples **a** without mini-G_S_ and **b** with mini-G_S_. **c** and **d** Ratio of transition rate constants between states 2 and 3 for samples **c** without mini-G_S_ and **d** with mini-G_S_. The vertical scales in panels **c** and **d** were expanded for clarity. See Supplementary Fig. 7 for a plot of panels **c** and **d** on the same scale as panels **a** and **b**. For all panels, error bars indicate the calculated standard deviation. The dotted horizontal lines indicate values of the rate constants equal to 1.

Given that the activation of A_2A_AR and its variants occurs sequentially through intermediate state 2, there was a compelling need to explore the impact of mutations and the presence of mini-G_S_ on the transitions between intermediate state 2 and state 1, as well as between states 2 and 3. Because a bi-exponential function was used to fit most of the observed transitions between states 1 and 2, we first determined an average rate constant for transitions between states 1 and 2, ${k}_{1\to 2}$ and for the inverse transition from state 2 to state 1, ${k}_{2\to 1}$ by weighting each rate with the observed number of transitions and determining the mean (see the “Methods” section for further details). The values for ${k}_{1\to 2}$ and for ${k}_{2\to 1}$ were then used to calculate the equilibrium rate-constant (${k}_{{{\rm {eq}}}({{{{\mathrm{1,2}}}}})}$), which we define as the ratio of ${k}_{1\to 2}$/${k}_{2\to 1}$ (Fig. 7a, b, and Supplementary Table 6). In the absence of mini-G_S_, the values of ${k}_{{{\rm {eq}}}({{{{\mathrm{1,2}}}}})}$ ranged from ~0.3 to ~3.2 across all A_2A_AR variants (Fig. 7b). For A_2A_AR, the addition of the agonist NECA increased the value of ${k}_{{{\rm {eq}}}({{{{\mathrm{1,2}}}}})}$ from ~1.2 to ~2.4, consistent with the idea that agonists increase the frequency of transitions to the intermediate state. The mutation D52N showed a clear impact on the values of ${k}_{{{\rm {eq}}}({{{{\mathrm{1,2}}}}})}$, which are reduced to <1 for all A_2A_AR[D52N] samples relative to A_2A_AR, indicating this mutation decreases the frequency of transitions to state 2 from state 1 (Fig. 7a).

The addition of mini-G_S_ increased the values of ${k}_{{{\rm {eq}}}({{{{\mathrm{1,2}}}}})}$ for most samples of A_2A_AR and A_2A_AR variants. Specifically, the addition of mini-G_S_ increased ${k}_{{{\rm {eq}}}({{{{\mathrm{1,2}}}}})}$ for apo A_2A_AR from ~1.2 to ~2.0, and for agonist-bound A_2A_AR from ~2.4 to ~3.8. The broadly-observed increase in ${k}_{{{\rm {eq}}}({{{{\mathrm{1,2}}}}})}$ is consistent with the idea that the addition of mini-G_S_ shifts the receptor conformational equilibrium away from an inactive conformation to more frequently populate the intermediate state. The clear exception to this observation is for A_2A_AR[D52N], where no increase in ${k}_{{{\rm {eq}}}({{{{\mathrm{1,2}}}}})}$ was observed for apo A_2A_AR[D52N] or in a complex with NECA (Fig. 7b). The reduction in the frequency of transitions to state 2 for A_2A_AR[D52N] is consistent with the known low G protein signaling for this variant^42^ and further confirms the importance of state 2 as critical to the function of active A_2A_AR. Both A_2A_AR[I92N] and A_2A_AR[R291Q] showed an increase in ${k}_{{{\rm {eq}}}({{{{\mathrm{1,2}}}}})}$ for complexes with the agonist NECA relative to the apo protein or antagonist-bound receptor (Fig. 7a). A_2A_AR[I92N] showed a modest increase in ${k}_{{{\rm {eq}}}({{{{\mathrm{1,2}}}}})}$ for the apo protein and agonist-bound receptor in the presence of mini-G_S_ (Fig. 7b) and the ${k}_{{{\rm {eq}}}({{{{\mathrm{1,2}}}}})}$ for A_2A_AR[R291Q] did not show a significant change upon the addition of mini-G_S_ for the agonist complex but did show an increase for the complex with the antagonist ZM241385 (Fig. 7b).

Unlike the rate constants governing transitions between states 1 and 2, our reduced chi-squared analysis indicated that the rate constants for transitions between states 2 and 3 were better described by a mono-exponential fit rather than a bi-exponential fit (Supplementary Fig. 6 and Supplementary Tables 7–14). Thus, unlike states 1 and 2, no additional substates were observed for state 3. For nearly all samples, the rate constants from states 2 to 3, ${k}_{2\to 3}$ were similar to the rate constants for the reverse process, ${k}_{3\to 2}$. Defining ${k}_{{{\rm {eq}}}({{{{\mathrm{2,3}}}}})}$ as the ratio of ${k}_{2\to 3}$/${k}_{3\to 2}$, we found that the range of values of ${k}_{{{\rm {eq}}}({{{{\mathrm{2,3}}}}})}$ for samples prepared without mini-G_S_ spanned from ~0.5 to ~1.5, with most values near 1 and the lowest value of 0.5 for apo A_2A_AR (Fig. 7c and Supplementary Table 5). The range of values of ${k}_{{{\rm {eq}}}({{{{\mathrm{2,3}}}}})}$ was significantly narrower than the range of values for ${k}_{{{\rm {eq}}}({{{{\mathrm{1,2}}}}})}$ for all receptors and for all sample preparations (Fig. 7a, b, and Supplementary Fig. 6). This indicates that the effect of mutations and of the addition of mini-G_S_ had a proportionally greater impact on the rate constants between states 1 and 2 compared with the rate constants between states 2 and 3.

## Discussion

Analysis of single-molecule fluorescence emission traces of human A_2A_AR in lipid nanodiscs revealed the presence of three distinct states both in the absence and presence of mini-G_S_, with no additional states observed upon the addition of mini-G_S_ (Figs. 2, 5, Supplementary Figs. 3 and 4). We are confident in concluding that no additional states were observed because in our analysis we observed unique states defined ~200 a.u. apart, well outside of the typical fluctuations observed that were not associated with different states, ±30–40 a.u., which is consistent with previous results^34^. Notably, the frequency of transitions to state 3 exhibited a significant increase in the presence of mini-G_S_. We also observed a significant increase in the relative area of emission state 3 for the A_2A_AR variants containing CAMs (Fig. 2 and Supplementary Table 2). These findings collectively indicate that the conformation of A_2A_AR in state 3 likely corresponds to the conformation of active A_2A_AR within the assembled ternary complex with mini-G_S_. Interestingly, this observation aligns with a recent NMR study demonstrating that complex formation with an agonist alone is adequate to induce a global conformation of A_2A_AR that resembles the conformation observed in a ternary complex^32^.

Concurrently, we observed identical relative areas of emission intensities of states 1 and 2 for both apo A_2A_AR and antagonist-bound A_2A_AR (Fig. 2 and Supplementary Table 2), indicating ZM241385 does not change the distribution of fluorescence emission states. This result appears in line with earlier ^19^F-NMR studies of A_2A_AR labeled with TET at position C289^26,29^ and also in line with single-molecule FRET observations measured on the sub-millisecond timescale^39^. In contrast, ^19^F-NMR studies of A_2A_AR labeled at position V229C showed an inverse agonist effect of ZM241385, which was attributed to a higher basal activity reported for samples from that study^25^. Interpretation of the data in the current study is in line with the relatively low basal activity reported for A_2A_AR^51^.

In the context of the agonist-bound A_2A_AR ternary complex, we have also noted the persistence of a finite occupancy for state 1. One possible implication of this observation is the potential for rapid dissociation and subsequent re-association of mini-G_S_, accompanied by corresponding conformational changes in the receptor. However, based on our experience, such events have not been observed in either the analytical characterization of A_2A_AR ternary complexes or in earlier NMR studies. An alternative interpretation of this data suggests the presence of multiple conformations of A_2A_AR within the assembled ternary complex, leading to varying fluorescence intensities. This interpretation aligns more closely with findings from studies of alternative GPCR conformations in ternary complexes, as observed in cryo-EM structures of a neurotensin receptor^53^ and β_2_AR^54^. The fluorescence probe employed in our study exhibits sensitivity to conformational changes pertinent to receptor function occurring at the intracellular surface. Analogous to spectroscopic methodologies like ^19^F-NMR, which utilize probes targeted at specific sites, there remains a possibility that subtle structural variations could be present in other receptor regions, such as the transmembrane domain or extracellular surface, which may not be directly detected by the probe. This also indicates that while the mean fluorescence intensity observed for state 2, referred to as the intermediate state, remains consistent across all samples, subtle structural variations in the intermediate state conformation could exist for different variants or in the absence and presence of the mini-G_S_ protein. Previously, we had proposed a similar hypothesis to rationalize potential structural differences between the intermediate state of agonist-bound and antagonist-bound A_2A_AR^34^.

**Fig. 8:**
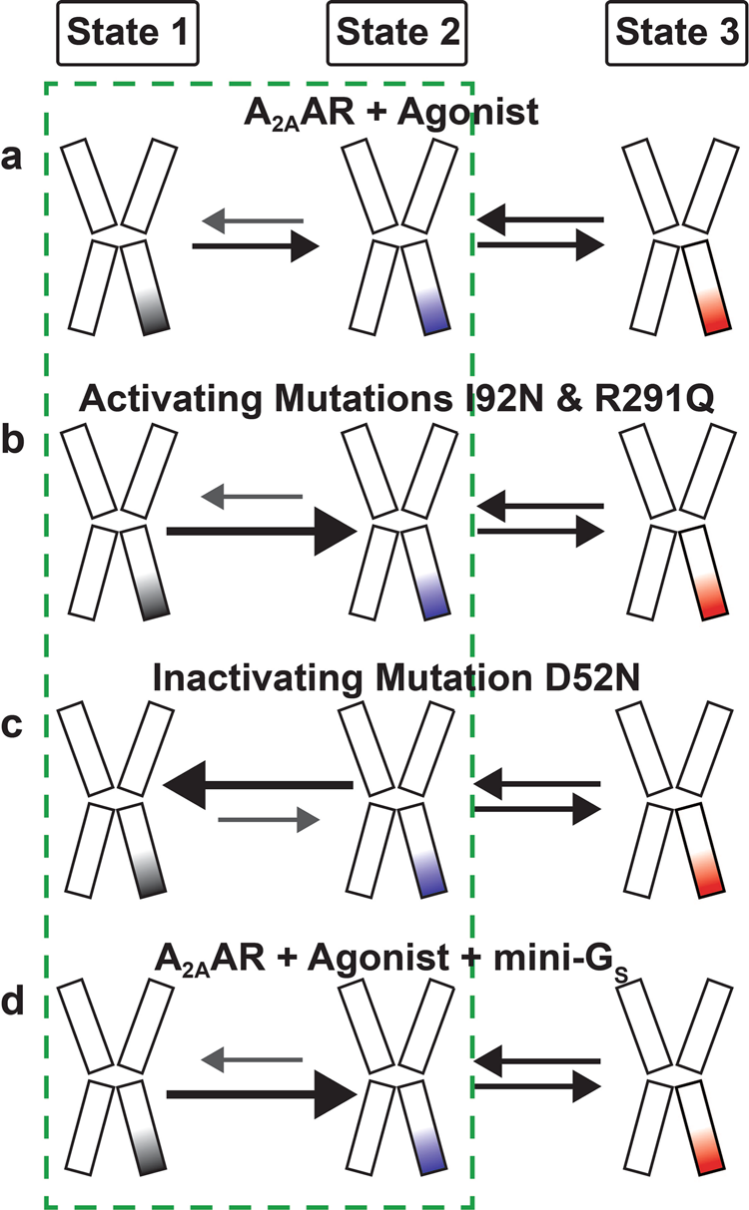
Visualization of the step-wise activation of A_2A_AR and influences on conformational equilibria. Schematic of the impact of agonists, mutations and presence of mini-G_S_ on the conformational equilibria describing A_2A_AR activation for **a** addition of agonists, **b** presence of activating mutations, **c** presence of inactivating mutations, and **d** addition of agonists and mini-G_S_. The relative sizes of the arrows indicate their preferred direction in the equilibrium. The dashed green box highlights the transitions between states 1 and 2, which showed the largest magnitude of differences among the observed samples.

The transition density plots shown in Fig. 3 provide clear evidence that CAMs do not increase the frequency of transitions between states 1 and 3. Instead, both the I92N and R291Q mutations increase the frequency of transitions to the intermediate state, state 2, as depicted in Fig. 8. It is notable that despite the I92N and R291Q mutations being located in distinct regions of the receptor, both mutations exert similar effects on the transition frequency to the intermediate state. These findings support the idea that both I92 and R291 are crucial elements within an allosteric pathway connecting the orthosteric drug binding site and intracellular surface. This interpretation aligns with previous NMR investigations involving R291^26^ and theoretical analysis involving I92^41^. Earlier investigations utilizing single-molecule fluorescence indicated that the activation of A_2A_AR by agonists followed a sequential and reversible step-wise process^34^. Findings from our current study align with this sequential activation model, reinforcing its consistency with visualizing the influence of CAMs and the addition of the partner mini-G_S_ protein.

Measurement of the mean occupancy times of A_2A_AR[I92N] and A_2A_AR[R291Q] revealed significantly reduced lifetimes of state 1 for these two variants, including for the apo receptors and their complexes with the antagonist ZM241385 (Supplementary Fig. 6). Concurrently, examination of the dwell time data of the same variants indicated an increased number of transitions from state 1 to state 2 for both A_2A_AR[I92N] and A_2A_AR[R291Q] (Fig. 7 and Supplementary Fig. 6). These findings align with the notion that constitutively activating mutations decrease the energy barrier between states 1 and 2 by raising the relative energy of state 1, thereby destabilizing it. For A_2A_AR, we observed a decreased mean occupancy time for state 1 for the agonist complex in the presence of mini-G_S_, suggesting that the addition of agonists and mini-G_S_ also increases the relative energy of state 1. Notably, the mean occupancy time of state 1 for A_2A_AR[D52N] remained relatively higher even in the presence of both an agonist and mini-G_S_ (Supplementary Fig. 6). Our observation of the longer mean occupancy time for A_2A_AR[D52N] appears consistent with the observation of attenuated conformational dynamics observed by NMR for the same variant^30^.

Analysis of the dwell time data for A_2A_AR and all A_2A_AR variants for transitions between states 1 and 2 required bi-exponential fits, consisting of both a slow transition and a fast transition. This indicated the presence of two sub-populations for both states 1 and 2, which each exhibited similar fluorescence emission intensities (Fig. 7). This observation resembles earlier single-molecule fluorescence studies of the β_2_AR, where analogous slow and fast transitions were observed between two fluorescence emission states^35,36^. It is intriguing to speculate whether similar observations will be reported for additional class A GPCRs in future studies.

Our findings underscore a significant advantage of single-molecule fluorescence in elucidating the mechanisms of GPCR signal transduction. Specifically, SMF allows one to determine the sequential order of molecular events involving multiple distinct protein conformations. Existing literature, particularly studies employing spectroscopic techniques such as NMR spectroscopy, has established a connection between the conformational equilibria of GPCRs and their signaling functions^55^. By confirming and complementing these observations, single-molecule fluorescence adds valuable information on the energy landscape of GPCR activation and signaling^33,38,39,56^. In our study, the use of single-molecular fluorescence enabled us to identify a crucial intermediate state essential for A_2A_AR activation. Furthermore, it facilitated the assessment of how the introduction of a G protein or the presence of activating or inactivating mutations influenced the conformational equilibria, favoring or inhibiting the population of the intermediate state (Fig. 8). Consequently, the functional impact of mutations on A_2A_AR can be directly linked to their effect on the frequency of transitions to the intermediate state.

Over 60 crystal and cryo-EM structures of human A_2A_AR have been publicly released, including complexes with antagonists, agonists, and ternary complexes with agonists and G proteins. Further, multiple crystal structures are available of A_2A_AR in complex with an agonist but not G protein, which have been proposed to be “active-like” or intermediate states^57,58^. Despite this, our modeling of single-molecule fluorescence data showed that a structure of this crucial intermediate state remains unknown^34^. The relationship between the population of the intermediate state and the A_2A_AR function suggests an innovative approach for testing or designing mutations with unique properties by measuring the impact of mutations on the intermediate state. Further structural elucidation of intermediate states may also present unexplored opportunities for targeted drug discovery.

## Methods

### Molecular cloning

The gene for human A_2A_AR (1–316) was cloned into a pPIC9K vector (Invitrogen) using the restriction sites BamHI and NotI. The receptor sequence included a single amino acid replacement to remove a putative glycosylation site (N154Q), an N-terminal FLAG tag, and a 10 X C-terminal HIS tag. This sequence was used as a template to generate the variants A_2A_AR[A289C], A_2A_AR[A289C,R291Q], A_2A_AR[A289C,I92N] and A_2A_AR[A289C,D52N] using site-directed mutagenesis with the Accuprime *Pfx* SuperMix (Thermo Fisher Scientific) and primers listed in Supplementary Table 1.

### Transformation to *Pichia pastoris* and colony selection

Recombinant pPIC9K plasmids containing A_2A_AR variants were transformed into the *Pichia pastoris* strain BG12 (Biogrammatics) using electroporation. For each variant, 20–25 colonies were selected and plated individually to identify high-expressing clones using a Western blot assay^28,30,43^. Glycerol stocks of colonies expressing the highest levels of receptor were prepared and stored at −80 °C.

### A_2A_AR expression and purification

All A_2A_AR variants were expressed using protocols reported in earlier studies^30,43^. For each expressed A_2A_AR variant, 400 µL of a glycerol stock was used to inoculate 4 mL of buffered minimal glycerol (BMGY) media. The cultures were incubated at 30 °C for 48 h, shaking at 200 RPM. After 48 h, the 4 mL cultures were used to inoculate 50 mL of BMGY media. These cultures were allowed to grow at 30 °C for 60 h shaking at 200 RPM. The 50 mL cultures were used to inoculate 500 mL of BMGY media, and the 500 mL cultures were allowed to grow for 48 h at 30 °C, shaking at 200 RPM. After 48 h, the 500 mL cultures were centrifuged at 3000 × *g* for 15 min, the supernatant was discarded and the pellets were resuspended in 500 mL fresh buffered minimal methanol (BMMY) media without methanol. To remove trace amounts of glycerol remaining in the cultures, the cells were allowed to grow for 6 h at 28 °C and 200 RPM shaking. Subsequently, aliquots of 0.5% w/v methanol were added to induce protein expression three times at 12-h intervals. 12 h after the last induction, the cultures were centrifuged at 3000 × *g* for 15 min, and the cells were harvested, frozen, and stored at −80 °C for future use.

Cell pellets were resuspended in buffer (50 mM sodium phosphate pH 7.0, 100 mM NaCl, 5% glycerol (w/v), and in-house prepared protease inhibitor cocktail solution) and lysed via two passes through a cell disrupter (Pressure Biosciences) operating at 40 PSI. Insoluble fractions from the lysed cells were centrifuged at 200,000 × *g* for 30 min, the solution was decanted, and the pellets were stored at −80 °C.

To purify A_2A_AR variants, membrane pellets were resuspended in buffer (10 mM HEPES pH 7.0, 10 mM KCl, 20 mM MgCl_2_, 1 M NaCl) and centrifuged at 200,000 × *g*. Then the pellets were resuspended in the same buffer containing 1 mM theophylline and protease inhibitor and incubated for 30 min at 4 °C. Resuspended membranes were mixed in a ratio of 1:1 with solubilizing buffer (50 mM HEPES pH 7.0, 500 mM NaCl, 0.5% (w/v) n-dodecyl-β-d-maltopyranoside (DDM), and 0.05% cholesteryl hemisuccinate (CHS)) for 6 h at 4 °C. Insoluble material was removed by ultracentrifugation at 200,000 × *g* for 30 min. The resulting solution containing solubilized receptors was treated with Co^2+^-charged affinity resin (Talon, Clontech) and 30 mM imidazole and incubated at 4 °C with gentle rotation. The resin was then washed with 25 column volumes of wash buffer 1 (50 mM HEPES pH 7.0, 500 mM NaCl, 10 mM MgCl_2_, 30 mM imidazole, 8 mM ATP, 0.05% DDM, and 0.005% CHS) followed by 20 column volumes of wash buffer 2 (25 mM HEPES pH 7.0, 250 mM NaCl, 5% glycerol, 30 mM imidazole, 0.05% DDM, 0.005% CHS, and an excess of ligand). A_2A_AR variants were eluted in a third buffer (50 mM HEPES pH 7.0, 250 mM NaCl, 5% glycerol, 300 mM imidazole, 0.05% DDM, 0.005% CHS, and ligand). Following this, samples were exchanged into buffer 4 (25 mM HEPES pH 7.0, 75 mM NaCl, 0.05% DDM, 0.005% CHS, and ligand) using a PD-10 desalting column (Cytiva) equilibrated with buffer 4. Saturating concentrations of ligands were used for buffers containing ligands. For apo samples, no ligand was added to any of the buffers.

### Nanodisc assembly

Nanodisc assembly was carried out according to protocols from earlier studies^59,60^. For expression and purification of the scaffold protein MSP1D1, a single colony of *E. coli* BL21(DE3) containing MSP1D1 in pET28a vector was used to inoculate 10 mL LB medium with 50 μg/mL Kanamycin. After 12–14 h of incubation at 37 °C with shaking at 200 RPM, this culture was used to inoculate 1 L Terrific Broth (TB) medium containing kanamycin in a baffled flask and was incubated at 37 °C with shaking at 200 RPM until the optical density (OD) reached 0.6–0.8. MSP1D1 expression was induced by adding 1 mM final concentration of isopropyl β-d-1-thiogalactopyranoside (IPTG) to the cells, and incubation continued for 4 h at 37 °C. Cells were harvested by centrifuging at 4000×*g* for 30 min at 4 °C. For lysis, cells were resuspended in MSP lysis buffer 1(50 mM Tris–HCl, pH 8.0, 500 mM NaCl, 1 mM EDTA, 1% triton X-100, and in-house protease inhibitor solution) and passed through a cell disrupter (Pressure Biosciences) operating at 20,000 PSI. Lysed cells were pelleted at 20,000 × *g* for 40 min. The supernatant was added to Ni-NTA resin pre-equilibrated with wash buffer 2 (50 mM Tris–HCl, pH 8.0, 500 mM NaCl, and 1% (w/v) Triton X-100), and the mixture was incubated at 4 °C with rotation for 2 h. The resin was washed with 5 CV wash buffer 2 followed by 5 CVs wash buffer 3 (50 mM Tris–HCl, pH 8.0, 500 mM NaCl, and 50 mM cholate), 5 CVs of wash buffer 4 (50 mM Tris–HCl, pH 8.0, and 500 mM NaCl) and finally 5 CVs of wash buffer 5 (50 mM Tris–HCl, pH 8.0, 500 mM NaCl, and 20 mM imidazole). MSP1D1 was eluted in buffer (50 mM Tris–HCl, pH 8.0, 500 mM NaCl, and 500 mM imidazole) and then dialyzed in a 10 kDa MWCO dialysis tube using buffer 7 (50 mM Tris–HCl, pH 8.0, 20 mM NaCl, and 0.5 mM EDTA). TEV protease was then added to MSP1D1 in a 1:100 ratio (w/w), and the solution was incubated overnight at 4 °C. The next morning, Ni-NTA was added to the solution and the mixture was run through a gravity column. MSP1D1 was collected as the flow-through. MSP1D1 was then dialyzed again in buffer 8 (20 mM Tris–HCl, pH 8.0, 100 mM NaCl, and 0.5 mM EDTA) for 4 h at 4 °C. MSP1D1 was concentrated to 1 mM and aliquoted stocks were stored at −80 °C for future use.

To prepare the stock solutions of lipids, dried lipids were dissolved in cholate buffer (25 mM Tris–HCl, pH 8.0, 150 mM NaCl, and 200 mM sodium cholate) to a concentration of 100 mM. A_2A_AR (27 μM in DDM/CHS mixed micelles), lipids, and MSP1D1 were combined at a molar ratio of 1:5:250 and incubated for 1–2 h at 4 °C with gentle rotation. After incubation, pre-washed bio-beads (Bio-Rad Laboratories) were added, and the mixture was incubated overnight at 4 °C. Following the overnight incubation, the bio-beads were removed from the solution and Ni-NTA resin (GoldBio) was added and incubated with the mixture for 24 h at 4 °C. Following this, the resin was washed with 2 CVs of nanodisc wash buffer (50 mM HEPES, pH 7.0, 150 mM NaCl, and 10 mM imidazole), and nanodiscs containing A_2A_AR were eluted with nanodisc elution buffer (50 mM HEPES, pH 7.0, 150 mM NaCl, 300 mM imidazole and ligand). Samples were then exchanged into a final buffer containing 25 mM HEPES pH 7.0, 75 mM NaCl, and excess ligand.

### Mini-G_S_ expression and purification

Human mini-G_S_ containing an N-terminal 6 X HIS tag and TEV protease recognition sequence was expressed and purified according to protocols from previous publications^49,61^. A single colony of the *E.coli* strain BL21(DE3)RIL containing mini-G_S_ in a pET28a vector was inoculated in 4 mL LB media supplemented with 0.2% glucose, 34 μg/mL chloramphenicol and 100 μg/mL carbenicillin and incubated at 37 °C for 8 h. This culture was used to inoculate 75 mL of the same media. The cells were allowed to grow at 30 °C overnight shaking at 200 RPM. The next morning, the cells were harvested and resuspended in 1 L M9 minimal media and allowed to grow at 30 °C, with 200 RPM shaking for 5–6 h until the OD reached ~0.8. To induce the protein expression, 50 μM IPTG was added to the culture and the cells were incubated at 25 °C for 16 h with 200 RPM shaking. Cells were then harvested and stored at −80 °C for future use.

Cells were lysed in buffer (25 mM Tris–HCl, pH 8.0, 150 mM NaCl, 1 mM MgCl_2_, 5 μM GDP, protease inhibitor) with a cell disruptor (Pressure Biosciences) operating at 22 kPSI. The lysate was pelleted at 35,000×*g* for 30 min. The supernatant was collected and incubated with Ni-NTA resin overnight at 4 °C. The next morning, the resin was washed with 20 CVs of wash buffer (25 mM Tris–HCl pH 8.0, 500 mM NaCl, 1 mM MgCl_2_, 5 mM imidazole, 5 μM GDP, and 2 mg/mL iodoacetamide). The protein was then eluted with buffer (25 mM Tris–HCl pH 8.0, 250 mM NaCl, 1 mM MgCl_2_, 250 mM imidazole, 10% (v/v) glycerol, and 5 μM GDP) and exchanged into a different buffer (25 mM Tris–HCl pH 8.0, 100 mM NaCl, 1 mM MgCl_2_, 10% (v/v) glycerol, 5 μM GDP). TEV protease was added to the solution at a 1:50 ratio (TEV: Mini-Gs) and incubated for ~16 h at 4 °C. Following this, Ni-NTA resin was added and incubated with the protein. The resin was washed with additional buffer and cleaved mini-G_S_ was collected as the flow through. Mini-G_S_ was further purified using gel-filtration with buffer (20 mM HEPES pH 8.0, 150 mM NaCl, 5 mM MgCl_2_, 1 mM EDTA, 10% (v/v) glycerol, and 5 μM GDP). Purified fractions containing the protein were concentrated to 50 μM, frozen, and stored at −80 °C for future use.

### A_2A_AR-Mini-G_S_ complex formation

Formation of the A_2A_AR-mini-G_S_ ternary complex was carried out by adapting protocols described in earlier publications^49,61^. Nanodiscs containing A_2A_AR variants were combined with a 1.3-fold molar excess of mini-G_S_, 1 mM MgCl_2,_ and apyrase (0.2 U) and incubated 4–6 h at 4 °C.

### A_2A_AR single molecular TIRF experiment

The A_2A_AR-nanodisc complexes containing 5% biotin-PE were immobilized on polyethylene glycol (m-PEG-SVA and 3% biotin-PEG-SVA) passivated quartz slides using biotin–streptavidin interactions. Detailed steps describing the cleaning and PEG passivation of the slides were described in our previous studies^34,43^. First, the surface of slides and coverslips was activated by 1 M potassium hydroxide solution, and silanized by incubating them in a 3% aminopropyltriethoxysilane (APTES) solution. The silanized surfaces were passivated with mPEG-SVA and Biotin-PEG-SVA solution prepared in 100 mM sodium bicarbonate buffer in the dark overnight. Finally, slides and coverslips were rinsed with deionized water, dried with nitrogen, and stored at −20 °C.

Fluorescence time traces were recorded for Cy3-labeled A_2A_AR, A_2A_AR[D52N], A_2A_AR[I92N], and A_2A_AR[R291Q] samples with no ligand added (apo) and for complexes with the antagonist ZM241385 and the agonist NECA. We used a customized prism-based TIRF imaging system based on an inverted IX73 microscope (Olympus) and customized TIRF stage (TIRF Labs Inc., Cary, NC, USA) for data acquisition^34,43^. Double-sided tape was used to prepare the flow chamber on the PEGylated quartz surface, then Cy3-labeled A_2A_AR-nanodisc complexes were applied and tethered to the surface by biotin and streptavidin interactions. After sample immobilization, the sample chamber was washed with imaging buffer (25 mM HEPES pH 7.5, 75 mM NaCl, 2 mM Trolox, and saturating concentration of ligand or no ligand added) and flushed with glucose oxidase and a catalase oxygen scavenging system (OSS) to increase the photostability of the dye. For TIRF experiments with mini-G_S_, the preformed ternary complex was diluted in imaging buffer containing 1 mM MgCl_2_, apyrase (0.2 U), and mini-G_S_, washed and flushed with 50 μM ligand and an additional 12.5 nM mini-Gs in OSS-containing imaging buffer with 1 mM MgCl_2_ and apyrase (0.2 U) to guarantee the stability of the complex. The immobilized Cy3-labeled samples were excited by a green laser (532 nm), and the emission signals were collected on an EMCCD camera (Andor Technology) in real-time^34,43^.

Single-molecule data acquisition and visualization programs were used to record single-molecule videos and process single-molecule time trajectories (https://github.com/Ha-SingleMoleculeLab/smFRET-Quickstart-Guide). We selected the dynamic single-molecule traces with single-step photobleaching and normalized them with the lowest intensity state for further analysis. We then use the ‘maximum-likelihood Hidden Markov model (HMM)’ fitting to distinguish the states^34,48^.

### Histogram analysis

For the histogram analysis in each condition, the normalized population intensities in the histograms were fit to Gaussian distributions using the Multipeak Fit 2 function in the Igor Pro software (version 8.04, Wavemetrics, Lake Oswego, OR, USA)^34,43^. In detail, we used the “Find Peaks” algorithm in Igor Pro to locate the number of peaks and fit those peaks using the mean conformational states as peak centers as determined from analysis of the transition density plots (TDP). The TDPs were generated by a custom-written script in MATLAB (Figs. 3 and 6). The peak position centers are better defined by the TDP analysis because they are better resolved in these 2-dimensional correlations. We then revisited the histogram analysis by using the peak center values determined from the TDP as input and allowing all other parameters to vary. We then used Igor’s “Multipeak Fit 2” function, $y(x)={y}_{0}+\mathop{\sum }\nolimits_{i=2}^{3}{A}_{i}\cdot {\rm {{e}}}^{{-(\frac{x-{\mu }_{i}}{{\sigma }_{i}})}^{2}}$, to fit the Gaussian distributions. In this equation, ${y}_{0}$ is the baseline, $i$ is the number of the Gaussian peaks (*i* = 2 or 3), ${A}_{i}$ is the peak height or amplitude, ${\mu }_{i}$ is the peak center and$,$ ${\sigma }_{i}$ is the peak width. The goodness of the fit was evaluated by a Chi-Squared analysis. The area of individual peaks was calculated from the ${A}_{i}$ and ${\sigma }_{i}$ values obtained from the fit.

### Dwell time analysis

The TDP shown in Figs. 3 and 6 are used to define the cut-off values. These values were then used to separate the dwell time data generated by the HaMMy code into the corresponding state transitions (e.g., state 1 to 2, state 1 to 3, etc.). The dwell times for each state transition were then plotted as histograms whose bin widths were calculated by the Freedman–Diaconis rule, ${{{\mbox{bin}}} \, {{\mbox{width}}}}=2\cdot {{IQR}}(x)/\root 3 \of {n}$, where ${{\rm {IQR}}}(x)$ is the interquartile range of the sample data $x$ and $n$ is the number of observations in the data^62^. Then, the histograms were fitted by both the mono-exponential and bi-exponential decay fits whose equations were given by, ${A\cdot {\rm {e}}}^{-k\cdot x}$ and ${A}_{1}\cdot {\rm {{e}}}^{-{k}_{1}\cdot x}+{A}_{2}\cdot {\rm {{e}}}^{-{k}_{2}\cdot x}$ respectively where *A*, *A*_1_ and *A*_2_ are the amplitudes of the fit, and *k*$,$ *k*_1_ and *k*_2_ are the rate constants calculated from the fit. The amplitude values in the dwell-time analysis correspond to the number of transitions between each state (for example, ${A}_{1}$ is the number of transitions with the rate constant ${k}_{1}$). To analyze the bi-exponential fit, we normalized the two amplitude values and converted them to percentages, such that ${A}_{1}+{A}_{2}=100 \%$ (Supplementary Tables 7–14). The goodness of fits was evaluated by reduced chi-square analysis. The histograms, corresponding fits, and chi-square test results were performed using Igor Pro software (version 8.04, Wavemetrics, Lake Oswego, OR, USA). In the case of bi-exponential fits, the two fit constants, ${k}_{1},$ and ${k}_{2}$ are averaged by the corresponding amplitudes ${A}_{1}$ and ${A}_{2}$, and is given by, $1/k={A}_{1}/{k}_{1}+{A}_{2}/{k}_{2}$^63^$.$ Based on the chi-squared test results for different conditions, we selected either mono-exponential or bi-exponential fits and used them for the final calculation of equilibrium rate-constant ratio ${k}_{{{\rm {eq}}}(i,j)}$ (ratio of the rate constant from state $i$ to state $j$ to the rate constant from state $j$ to state $i$) for each state transition. The equilibrium rate-constant ratio for each condition and each state-transition are shown in Fig. 7, Supplementary Figs. 5–7, and are tabulated in Supplementary Tables 6–14.

### Radioligand binding experiments

Radioligand binding assays were performed with the same samples of A_2A_AR and A_2A_AR variants used for single-molecule fluorescence experiments. For all competition binding experiments, increasing concentrations of cold ligands (NECA or ZM241385) were incubated with 0.5 nM of radioligand ([^3^H]ZM241385 (American Radiolabeled Chemicals, St. Louis, MO) or [^3^H] CGS21680 (Perkin Elmer, Waltham, MA)) and with 0.125–0.25 µg of nanodiscs containing A_2A_AR in buffer (25 mM HEPES pH 7.0 and 75 mM NaCl) for 60 min at room temperature. Binding reactions were terminated by filtration using the Microbeta filtermat-96 cell harvester (PerkinElmer, Waltham, MA), and radioactivity was measured using a MicroBeta2 microplate counter (PerkinElmer, Waltham, MA). Specific binding at A_2A_AR was determined as the difference in binding obtained in the absence and presence of 10 µM ZM241385. IC_50_ values were determined using a nonlinear, least-square regression analysis in Prism 8 (GraphPad Software, Inc.). The *K*_D_ or *K*_I_ values were calculated from the IC_50_ using the Cheng−Prusoff equation^64^. Error bars were calculated as the standard error of the mean (s.e.m.) *n* ≥ 3 independent trials.

### Statistics and reproducibility

Statistical analysis of the competition binding experiments shown in Supplementary Fig. 2 was performed using Prism 8 (GraphPad Software, Inc.). The error bars shown in this figure are the standard error of the mean from *n* ≥ 3 independent trials, and the IC_50_ values were determined using a nonlinear, least-square regression analysis. The rest of the statistical analyses were performed using Igor Pro software (version 8.04, Wavemetrics, Lake Oswego, OR, USA). The error bars of the mean occupancy times (Supplementary Fig. 6A and B and Supplementary Table 5) are the standard error of the mean, and the error bars in the rest of the figures are the standard deviation. Multi-peak Gaussian fitting was used for normalized intensity histogram analysis (Figs. 2 and 5). For dwell time analysis (Fig. 7, Supplementary Figs. 5–7, Supplementary Tables 6–14), we used mono- and bi-exponential fitting along with chi-square analysis to test the goodness of fit.

### Reporting summary

Further information on research design is available in the Nature Portfolio Reporting Summary linked to this article.

### Supplementary information

Supplementary Information

Description of Supplementary Materials

Supplementary Data 1

Reporting Summary

## Data Availability

Previously published structures from the PDB can be accessed via accession codes 3EML and 5G53. Additional source data are provided with this paper as a Source Data (Supplementary Data 1) file. Further information and requests will be fulfilled by the corresponding authors.

## Code availability

The source code to generate TDP plots (Figs. 3 and 6) is available at: https://github.com/lamichhanelab/TDP_analysis.
