# Supplementary material for: Single-molecule visualization of human A2A adenosine receptor activation by a G protein and constitutively activating mutations
Source: Commun Biol. 2023 Nov 30;6:1218. doi: 10.1038/s42003-023-05603-6 (PMC10689853; doi:10.1038/s42003-023-05603-6)
Supplement: Supplementary file 1 — Supplementary Information [file 42003_2023_5603_MOESM1_ESM.pdf]

Supplementary Information

Single-Molecule Visualization of Human A_{2A} Adenosine Receptor Activation by a G Protein and Constitutively Activating Mutations

Shushu Wei[1], Niloofar Gopal Pour[2], Sriram Tiruvadi-Krishnan[1], Arka Prabha Ray[2], Naveen Thakur[2], Matthew T. Eddy[2*], Rajan Lamichhane[1*]

[1]Department of Biochemistry & Cellular and Molecular Biology, College of Arts and Sciences, University of Tennessee, Knoxville, USA

[2]Department of Chemistry, College of Liberal Arts and Sciences, University of Florida, Gainesville, USA

*Correspondence: matthew.eddy@ufl.edu, rajan@utk.edu

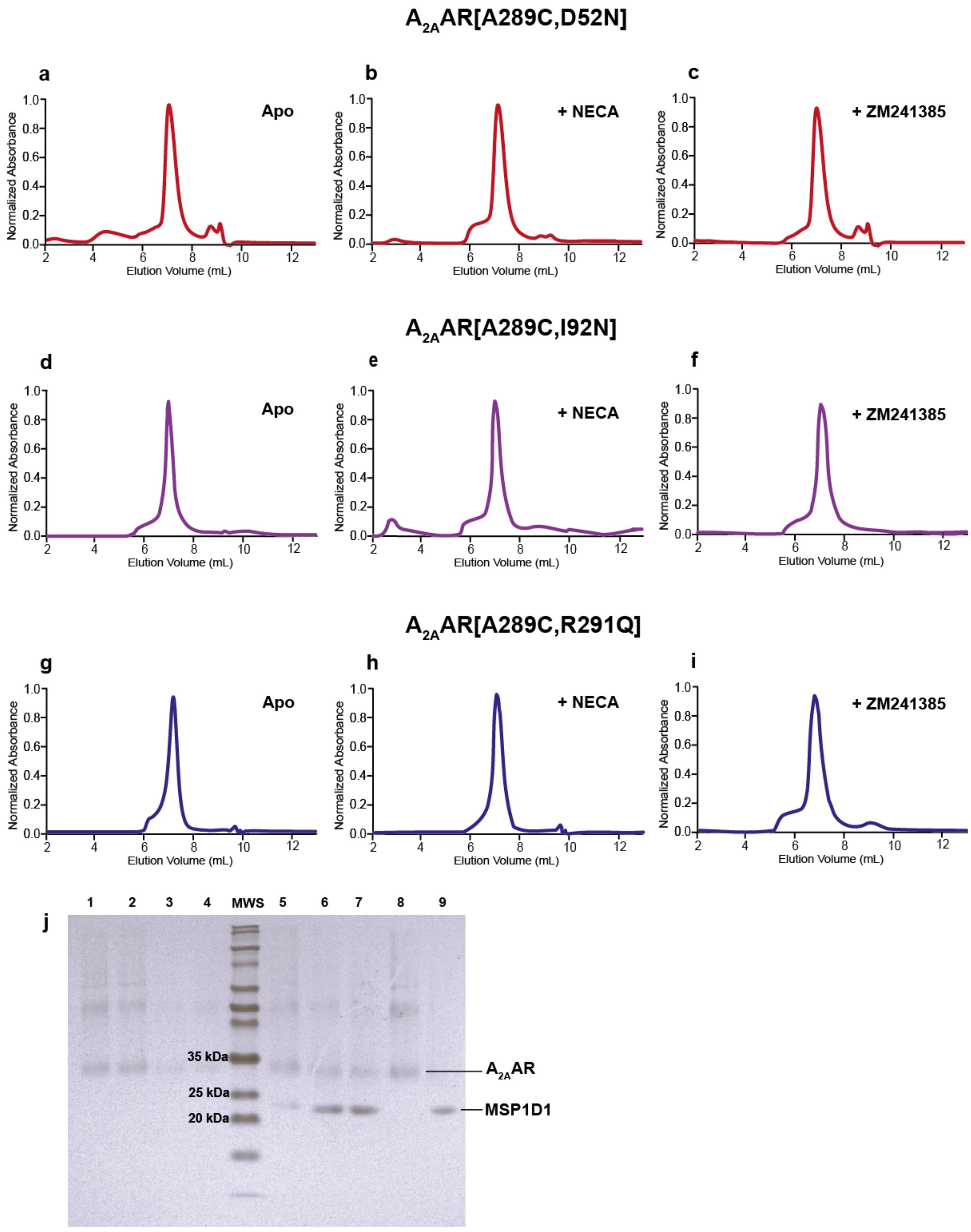

A2AAR[A289C,D52N]
a
Apo
b
+ NECA
c
+ ZM241385
Normalized Absorbance
Elution Volume (mL)
A2AAR[A289C,I92N]
d
Apo
e
+ NECA
f
+ ZM241385
A2AAR[A289C,R291Q]
g
Apo
h
+ NECA
i
+ ZM241385
j
1 2 3 4 MWS 5 6 7 8 9
35 kDa
25 kDa
20 kDa
A2AAR
MSP1D1

Supplementary Fig. 1. Analytical size exclusion chromatograms and SDS-PAGE image of purified A_{2A}AR[A289C] samples. (a-c) Size exclusion chromatograms for A_{2A}AR[A289C,D52N] without ligand added (apo) and in complexes with NECA or ZM241385 in lipid nanodiscs. (d-f) Size exclusion chromatograms for A_{2A}AR[A289C,I92N] without ligand added (apo) and in complexes with NECA or ZM241385 in lipid nanodiscs. (g-i) Size exclusion chromatograms for A_{2A}AR[A289C,R291Q] without ligand added (apo) and in complexes with NECA or ZM241385 in lipid nanodiscs. (j) SDS-PAGE image of purified apo A_{2A}AR[A289C] and the three apo A_{2A}AR variants in detergent and lipid nanodisc preparations, shown for lanes: (1) A_{2A}AR[A289C] in DDM/CHS micelles, (2) A_{2A}AR[A289C,D52N] in DDM/CHS micelles, 3 A_{2A}AR[A289C,I92N] in DDM/CHS micelles, (4) A_{2A}AR[A289C,R291Q] in DDM/CHS micelles, (5) A_{2A}AR[A289C,I92N] in lipid nanodiscs, (6) A_{2A}AR[A289C,R291Q] in lipid nanodiscs, (7) A_{2A}AR[A289C,D52N] in lipid nanodiscs, (8) A_{2A}AR[A289C] in lipid nanodiscs, (9) MSP1D1. "MWS" is the employed molecular weight standard.

Primer Name	Sequence (Oligonucleotide, 5’ to 3’)	Source
**A_{2A}AR_D52N_F**	TTTGTGGTGTCACTGGCGGCGGCCAACATCGCAGTGGGTGTGCTCGCCATCC	IDT
**A_{2A}AR_D52N_R**	GGATGGCGAGCACACCCACTGCGATGTTGGCCGCCGCCAGTGACACCACAAA	IDT
**A_{2A}AR_I92N_F**	GTCCTCACGCAGAGCTCCAACTTCAGTCTCCTGGCCATCGCCATTGACCG	IDT
**A_{2A}AR_I92N_R**	CGGTCAATGGCGATGGCCAGGAGACTGAAGTTGGAGCTCTGCGTGAGGAC	IDT
**A_{2A}AR_R291Q_F**	GTTGTGAATCCCTTCATTTACTGTTACCAAATCCGCGAGTTCCGCCAGAC	IDT
**A_{2A}AR_R291Q_R**	GTCTGGCGGAACTCGCGGATTTGGTAACAGTAAATGAAGGGATTCACAAC	IDT

Supplementary Table 1. Oligonucleotide sequences for generating A_{2A}AR variants from the A_{2A}AR[A289C] template plasmid.

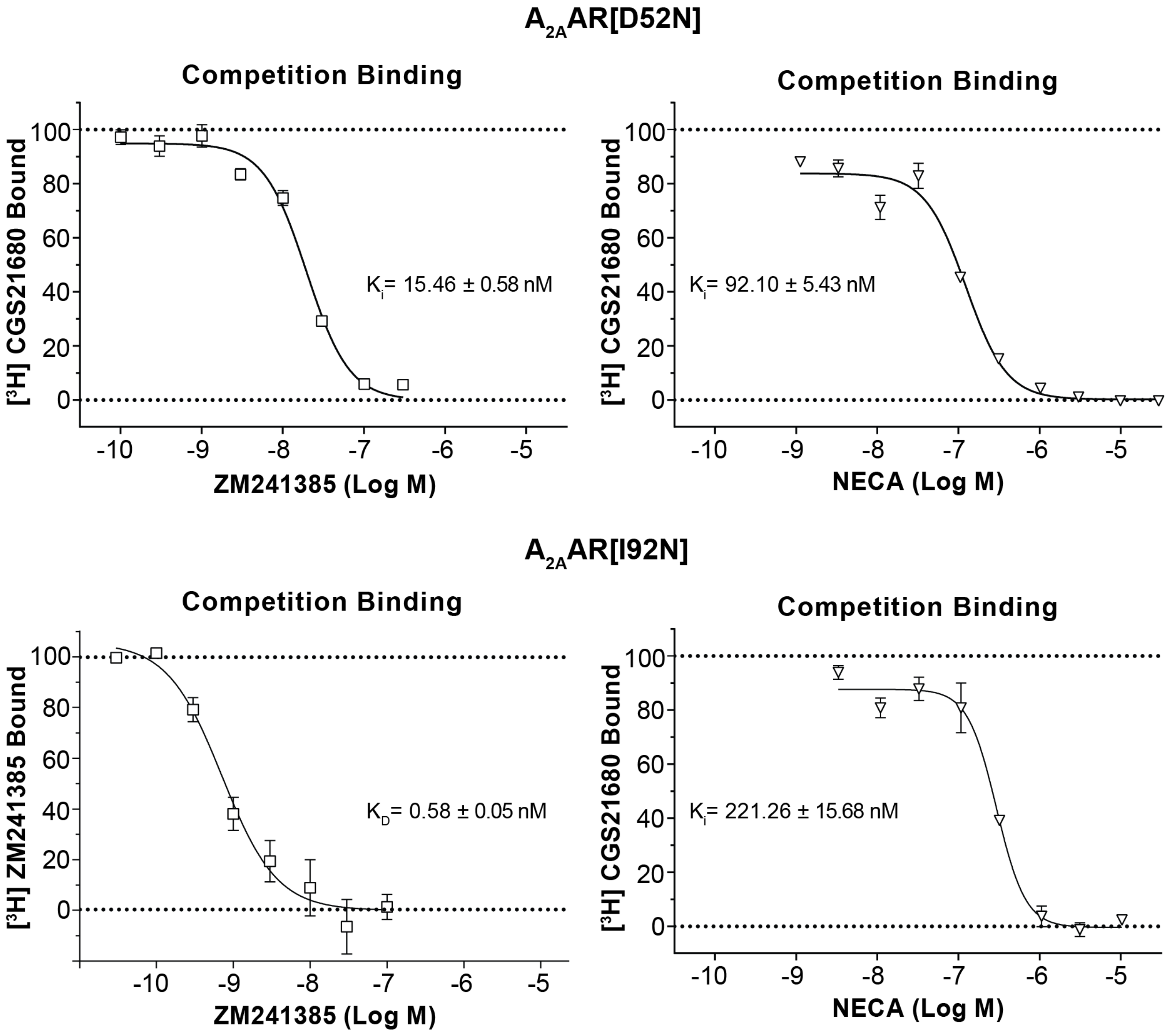

Supplementary Fig. 2. Pharmacological activity of A_{2A}AR[D52N] and A_{2A}AR[I92N] in lipid nanodiscs. Competition binding experiments with the antagonist ZM241385 are shown in the left panels, and competition binding experiments with the agonist NECA are shown in the right panels. The measured K_D or K_I values are shown in each panel. Error bars indicate the s.e.m for n $\geq$ 3 independent trials.

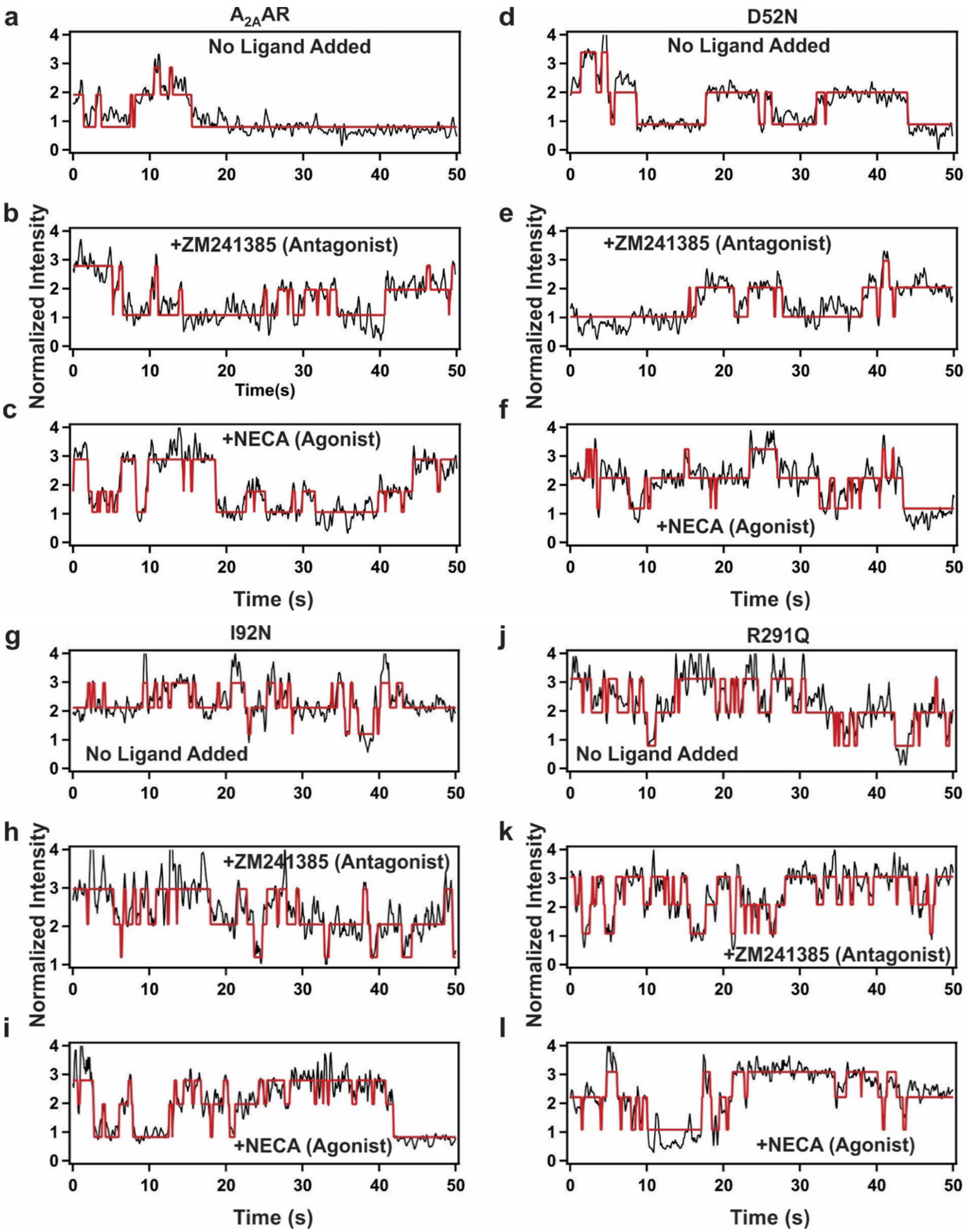

Supplementary Fig. 3. Dynamic single-molecule fluorescence time trajectories for (a-c) $A_{2A}AR$, (d-f) $A_{2A}AR$[D52N], (g-i) $A_{2A}AR$[I92N], and (j-l) $A_{2A}AR$[R291Q] in the absence of added ligand (apo) and for complexes with the antagonist ZM241385 and agonist NECA. The Cy3 intensity is normalized by the mean value of the lowest intensity state. The intensity trajectories (black) are fitted by a three-state hidden Markov model (red).

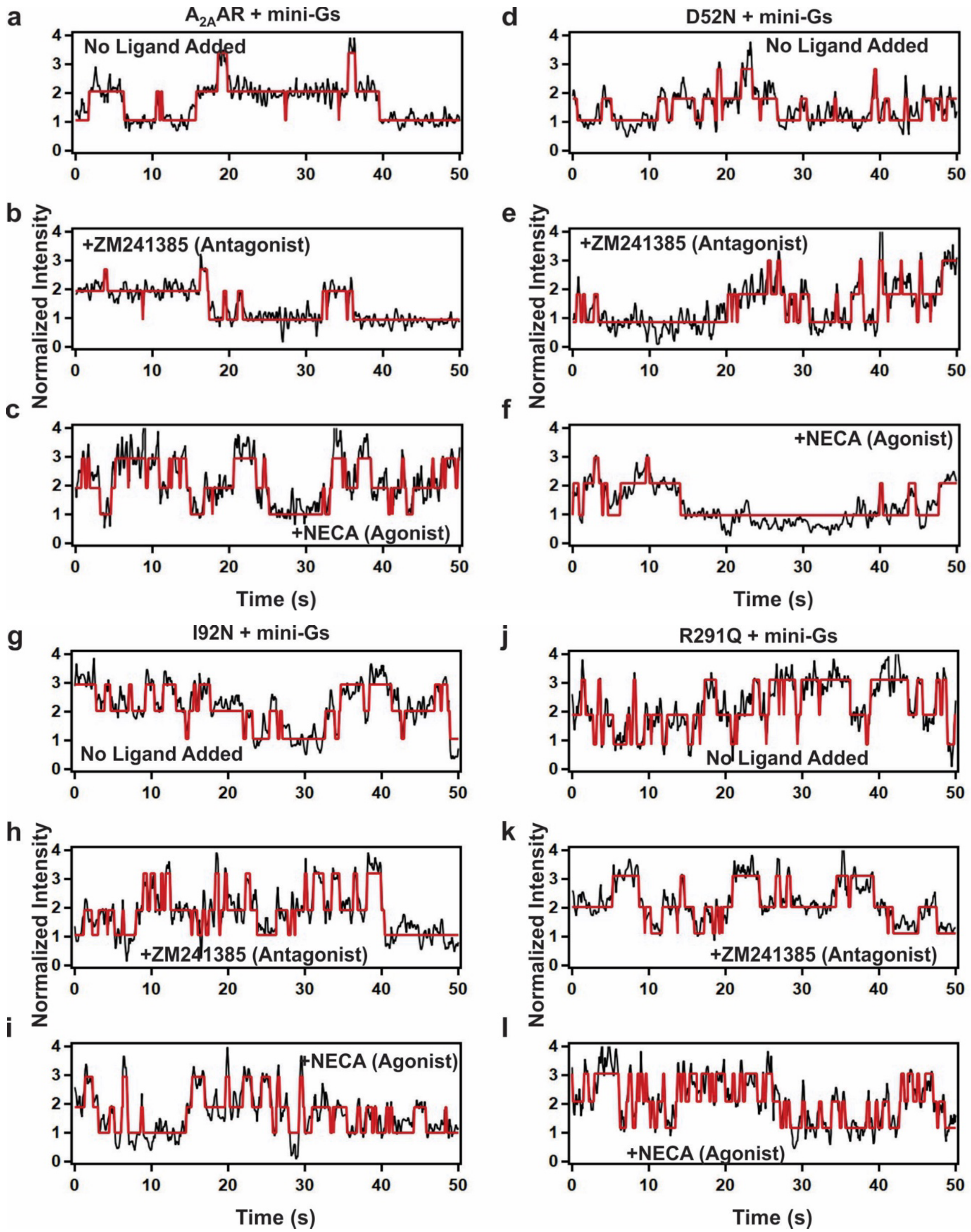

Supplementary Fig. 4. Dynamic single-molecule fluorescence time trajectories in the presence of mini-G_S for (a-c) A_{2A}AR, (d-f) A_{2A}AR[D52N], (g-i) A_{2A}AR[I92N], and (j-l) A_{2A}AR[R291Q] in the absence of added ligand (apo) and for complexes with the antagonist ZM241385 and agonist NECA. The Cy3 intensity is normalized by the mean value of the lowest intensity state. The intensity trajectories (black) are fitted by a three-state hidden Markov model (red).

Sample	$A_{2A}AR$ (%)			D52N (%)			I92N (%)			R291Q (%)		
	1	2	3	1	2	3	1	2	3	1	2	3
Apo	63 ± 1.1	37 ± 1.4	n/a	66 ± 2.5	26 ± 1.9	8 ± 3.2	39 ± 0.7	43 ± 0.7	18 ± 1.1	40 ± 0.7	45 ± 0.7	15 ± 1.0
ZM241385	63 ± 0.9	37 ± 1.1	n/a	60 ± 1.7	31 ± 1.3	9 ± 2.0	41 ± 1.0	40 ± 0.8	19 ± 1.1	34 ± 1.0	48 ± 0.9	18 ± 0.8
NECA	49 ± 0.7	37 ± 0.7	14 ± 1.0	59 ± 1.9	32 ± 1.4	9 ± 2.1	39 ± 1.0	41 ± 0.9	20 ± 1.4	33 ± 0.6	45 ± 0.6	22 ± 0.8

Sample	$A_{2A}AR$ + Gs (%)			D52N + Gs (%)			I92N + Gs (%)			R291Q + Gs (%)		
	1	2	3	1	2	3	1	2	3	1	2	3
Apo	54 ± 1.0	38 ±1.0	8 ± 1.4	64 ± 2.8	28 ± 2.1	7 ± 3.1	35 ± 0.6	47 ± 0.6	18 ± 0.8	30 ± 0.7	45 ± 0.6	25 ± 0.9
ZM241385	49 ± 1.0	43 ± 0.9	8 ± 1.1	66 ± 3.1	27 ± 2.4	7 ± 3.4	35 ± 1.0	47 ± 0.9	18 ± 1.3	28 ± 1.0	54 ± 0.8	18 ± 1.1
NECA	38 ± 1.0	41 ± 0.9	21 ± 1.2	62 ± 1.4	31 ± 1.1	7 ± 1.8	32 ± 0.6	48 ± 0.8	20 ± 1.0	33 ± 0.7	50 ± 0.6	17 ± 0.8

Supplementary Table 2. Relative areas of observed fluorescence emission intensity states, “1”, “2”, and “3” in the absence and presence of mini-G_S. Error bars indicate the standard deviation.

Sample	$A_{2A}AR$		D52N		I92N		R291Q	
	-Gs	+Gs	-Gs	+Gs	-Gs	+Gs	-Gs	+Gs
**Apo**	149	163	166	156	140	148	237	172
**ZM241385**	153	220	156	151	144	163	208	138
**NECA**	160	160	155	153	148	153	190	162

Supplementary Table 3. Number of dynamic molecules analyzed.

Sample	$A_{2A}AR$		D52N		I92N		R291Q	
	1 to 3	3 to 1	1 to 3	3 to 1	1 to 3	3 to 1	1 to 3	3 to 1
**Apo**	0.4%	0.4%	1.3%	1.5%	1.6%	2.1%	0.8%	1.5%
**ZM241385**	0.6%	0.9%	0.3%	0.5%	2.3%	3.0%	1.2%	1.7%
**NECA**	0.6%	0.9%	0.7%	1.4%	2.0%	2.5%	1.4%	2.7%
**Sample**	**$A_{2A}AR$ + Gs**		**D52N + Gs**		**I92N + Gs**		**R291Q + Gs**	
	1 to 3	3 to 1	1 to 3	3 to 1	1 to 3	3 to 1	1 to 3	3 to 1
**Apo**	0.5%	0.9%	0.8%	1.6%	2.2%	3.4%	1.9%	3.3%
**ZM241385**	1.1%	1.4%	1.3%	2.2%	1.7%	2.5%	1.3%	1.4%
**NECA**	0.7%	1.4%	1.2%	1.7%	2.2%	1.7%	1.3%	1.4%

Supplementary Table 4. Percentage of molecules transitioning between state 1 and state 3.

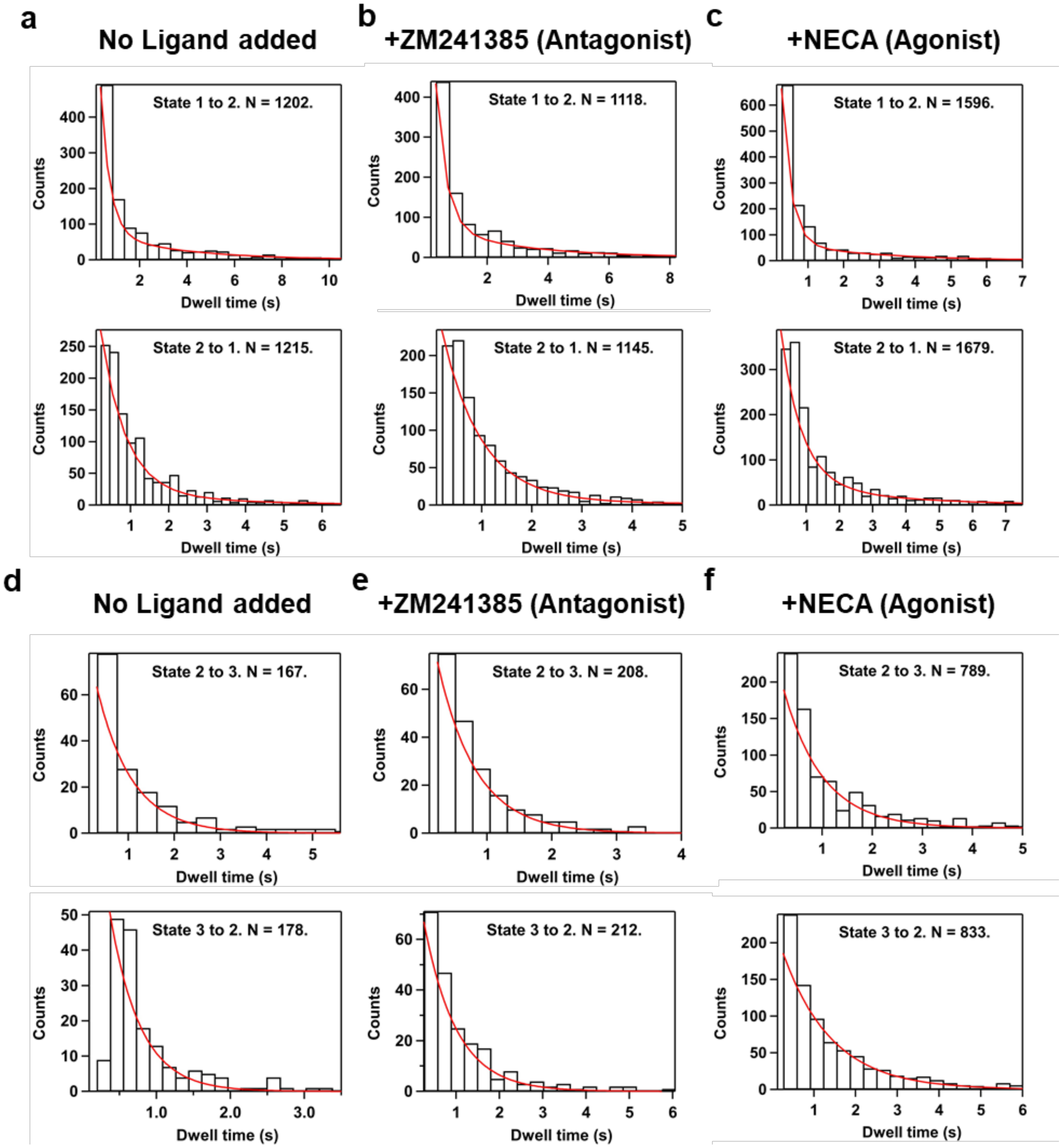

Supplementary Fig. 5. Dwell-time histograms. (a-c) Representative dwell-time histograms for transitions of $A_{2A}AR$ between state 1 and 2 are plotted for different conditions in the absence of mini-Gs. The solid red lines are the corresponding best fits to a bi-exponential function. (d-f) Representative dwell time histograms for transitions of $A_{2A}AR$ between state 2 and 3 are plotted for different conditions in the absence of mini-Gs. The solid red lines are the corresponding best fits to a mono-exponential function. “N” corresponds to the number of transitions analyzed.

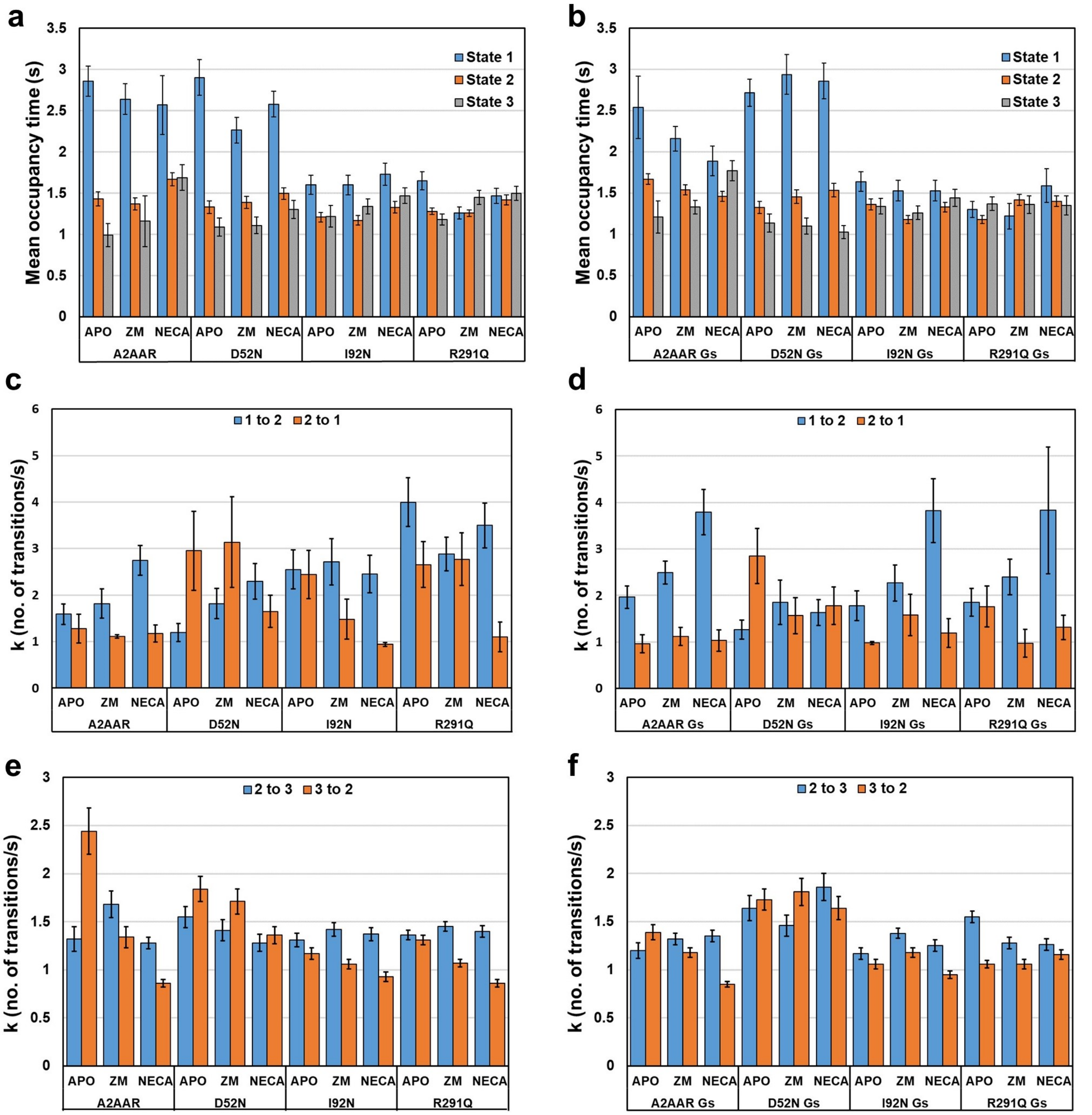

Supplementary Fig. 6. Dwell time analysis. (a and b) Mean occupancy times in states 1, 2 and 3 of $A_{2A}AR$ and its variants are plotted for different conditions in the absence (a) and in the presence (b) of mini-G_S. Error bars indicate the standard error of the mean. (c and d) The average rate constant k for transitions of $A_{2A}AR$ between states 1 and 2 are shown for various conditions in the absence (c) and in the presence (d) of mini-G_S. Error bars indicate the standard deviation. (e and f) The rate constant k for transitions of $A_{2A}AR$ between states 2 and 3 are shown for various conditions in the absence (e) and in the presence (f) of mini-G_S. Error bars indicate the standard deviation.

Sample	$A_{2A}AR$			D52N			I92N			R291Q		
	1	2	3	1	2	3	1	2	3	1	2	3
**Apo**	2.86 ± 0.18	1.43 ± 0.09	0.99 ± 0.14	2.90 ± 0.22	1.33 ± 0.08	1.09 ± 0.11	1.60 ± 0.12	1.21 ± 0.06	1.22 ± 0.13	1.65 ± 0.11	1.28 ± 0.04	1.18 ± 0.07
**ZM241385**	2.64 ± 0.19	1.37 ± 0.07	1.16 ± 0.31	2.26 ± 0.16	1.39 ± 0.07	1.11 ± 0.10	1.60 ± 0.12	1.17 ± 0.06	1.34 ± 0.09	1.26 ± 0.07	1.26 ± 0.04	1.45 ± 0.09
**NECA**	2.57 ± 0.36	1.67 ± 0.08	1.69 ± 0.16	2.58 ± 0.15	1.50 ± 0.07	1.30 ± 0.11	1.73 ± 0.13	1.33 ± 0.07	1.47 ± 0.09	1.47 ± 0.09	1.42 ± 0.06	1.50 ± 0.08
**Sample**	**$A_{2A}AR$ + Gs**			**D52N + Gs**			**I92N + Gs**			**R291Q + Gs**		
	1	2	3	1	2	3	1	2	3	1	2	3
**Apo**	2.54 ± 0.38	1.67 ± 0.06	1.21 ± 0.20	2.72 ± 0.16	1.33 ± 0.07	1.14 ± 0.11	1.64 ± 0.12	1.36 ± 0.07	1.34 ± 0.09	1.30 ± 0.10	1.18 ± 0.05	1.37 ± 0.08
**ZM241385**	2.16 ± 0.15	1.54 ± 0.06	1.33 ± 0.08	2.94 ± 0.24	1.45 ± 0.08	1.10 ± 0.10	1.53 ± 0.12	1.18 ± 0.05	1.26 ± 0.08	1.22 ± 0.16	1.42 ± 0.07	1.36 ± 0.10
**NECA**	1.89 ± 0.18	1.46 ± 0.06	1.77 ± 0.12	2.86 ± 0.22	1.54 ± 0.08	1.03 ± 0.08	1.53 ± 0.13	1.33 ± 0.06	1.44 ± 0.10	1.59 ± 0.21	1.40 ± 0.06	1.35 ± 0.11

Supplementary Table 5. Mean-occupancy times ± standard error of the mean in seconds of observed fluorescence emission intensity states, “1”, “2”, and “3” in the absence and presence of mini-G_S.

Sample	$A_{2A}AR$	D52N	I92N	R291Q
	**1↔2**	**1↔2**	**1↔2**	**1↔2**
**Apo**	1.24 ± 0.35	0.40 ± 0.13	1.04 ± 0.28	1.51 ± 0.34
**ZM241385**	1.64 ± 0.29	0.58 ± 0.21	1.83 ± 0.63	1.04 ± 0.25
**NECA**	2.34 ± 0.45	1.39 ± 0.37	2.61 ± 0.45	3.17 ± 1.02
**Sample**	**$A_{2A}AR$ + Gs**	**D52N + Gs**	**I92N + Gs**	**R291Q + Gs**
	**1↔2**	**1↔2**	**1↔2**	**1↔2**
**Apo**	2.05 ± 0.49	0.45 ± 0.12	1.82 ± 0.33	1.05 ± 0.31
**ZM241385**	2.22 ± 0.44	1.18 ± 0.42	1.44 ± 0.47	2.47 ± 0.86
**NECA**	3.68 ± 0.95	0.92 ± 0.26	3.21 ± 1.02	2.92 ± 1.19
**Sample**	**$A_{2A}AR$**	**D52N**	**I92N**	**R291Q**
	**2↔3**	**2↔3**	**2↔3**	**2↔3**
**Apo**	0.54 ± 0.08	0.84 ± 0.08	1.12 ± 0.08	1.04 ± 0.06
**ZM241385**	1.25 ± 0.15	0.82 ± 0.09	1.34 ± 0.09	1.36 ± 0.07
**NECA**	1.49 ± 0.10	0.94 ± 0.09	1.47 ± 0.11	1.63 ± 0.10
**Sample**	**$A_{2A}AR$ + Gs**	**D52N + Gs**	**I92N + Gs**	**R291Q + Gs**
	**2↔3**	**2↔3**	**2↔3**	**2↔3**
**Apo**	0.86 ± 0.08	0.95 ± 0.10	1.10 ± 0.08	1.46 ± 0.08
**ZM241385**	1.12 ± 0.07	0.81 ± 0.09	1.17 ± 0.07	1.21 ± 0.08
**NECA**	1.59 ± 0.09	1.13 ± 0.12	1.32 ± 0.08	1.09 ± 0.07

Supplementary Table 6. The equilibrium rate constant ratios (k_{eq} ± standard deviation) calculated for each state transition are tabulated.

States	A ± sd (%)		k ± sd (1/s)		Reduced χ^2
**A_{2A}AR—APO**					
**1 to 2**	100		0.90 ± 0.04		2.41
	92.3 ± 9.7	7.7 ± 0.9	2.54 ± 0.2	0.29 ± 0.02	0.63
**2 to 1**	100		1.14 ± 0.04		0.47
	93.7 ± 5.7	6.3 ± 2.4	1.55 ± 0.1	0.36 ± 0.06	0.27
**2 to 3**	100		1.32 ± 0.13		1.4
	95.0 ± 19.1	5.0 ± 4.0	2.1 ± 0.45	0.29 ± 0.18	0.43
**3 to 2**	100		2.44 ± 0.24		0.58
	99.3 ± 21.4	0.7 ± 0.9	2.7 ± 0.36	0.22 ± 0.32	0.51
**A_{2A}AR—NECA**					
**1 to 2**	100		1.98 ± 0.07		1.99
	95.4 ± 8.4	4.6 ± 0.5	3.87 ± 0.26	0.39 ± 0.02	0.6
**2 to 1**	100		0.92 ± 0.03		1.82
	89.0 ± 5.6	11.0 ± 2.2	1.63 ± 0.13	0.36 ± 0.03	0.94
**2 to 3**	100		1.28 ± 0.06		1.29
	82.2 ± 11.6	17.8 ± 4.7	3.13 ± 0.58	0.71 ± 0.08	0.73
**3 to 2**	100		0.86 ± 0.04		0.57
	67.2 ± 13.4	32.8 ± 7.0	2.66 ± 0.78	0.64 ± 0.06	0.28
**A_{2A}AR—ZM241385**					
**1 to 2**	100		0.92 ± 0.04		1.36
	91.5 ± 10.7	8.5 ± 1.2	2.91 ± 0.30	0.36 ± 0.03	0.45
**2 to 1**	100		1.11 ± 0.04		0.53
	97.9 ± 5.3	2.1 ± 1.0	1.30 ± 0.07	0.22 ± 0.06	0.34
**2 to 3**	100		1.68 ± 0.14		0.38
	73.0 ± 61.6	27 ± 74.8	2.50 ± 2.03	1.15 ± 0.88	0.35
**3 to 2**	100		1.34 ± 0.11		0.81
	94.8 ± 14.7	5.2 ± 14.9	1.67 ± 0.47	0.45 ± 0.66	0.7

Supplementary Table 7: Mono-exponential and bi-exponential fitting results from the dwell time analysis of A_{2A}AR without ligand added (apo) and with ligands in the absence of mini-G_S. “A” is the reported amplitude and “sd” the calculated standard deviation, as described in the Methods section.

States	A ± sd (%)		k ± sd (1/s)		Reduced χ^2
**$A_{2A}AR$[R291Q]—APO**					
**1 to 2**	100		1.18 ± 0.04		3.32
	93.3 ± 11.8	6.7 ± 0.5	6.57 ± 0.53	0.62 ± 0.03	1.04
**2 to 1**	100		1.05 ± 0.03		2.07
	88.2 ± 17.4	11.8 ± 1.2	4.19 ± 0.51	0.71 ± 0.03	1.04
**2 to 3**	100		1.36 ± 0.05		1.12
	72.4 ± 15.1	27.6 ± 18.2	2.25 ± 0.60	0.92 ± 0.18	0.97
**3 to 2**	100		1.31 ± 0.05		1.21
	91.9 ± 48.4	8.1 ± 1.2	6.07 ± 1.37	0.97 ± 0.06	0.42
**$A_{2A}AR$[R291Q]—NECA**					
**1 to 2**	100		1.75 ± 0.06		2.26
	93.0 ± 10.0	7.0 ± 0.8	5.22 ± 0.42	0.65 ± 0.04	0.63
**2 to 1**	100		0.94 ± 0.03		1.24
	84.0 ± 7.0	16.0 ± 7.6	1.43 ± 0.18	0.50 ± 0.08	0.91
**2 to 3**	100		1.40 ± 0.06		1.34
	84.3 ± 11.8	15.7 ± 3.3	3.66 ± 0.58	0.76 ± 0.07	0.55
**3 to 2**	100		0.86 ± 0.04		1.74
	89.1 ± 11.7	10.9 ± 13.2	1.11 ± 0.19	0.42 ± 0.17	1.59
**$A_{2A}AR$[R291Q]—ZM241385**					
**1 to 2**	100		1.61 ± 0.05		1.71
	89.4 ± 7.7	10.6 ± 1.1	4.49 ± 0.35	0.72 ± 0.04	0.94
**2 to 1**	100		1.07 ± 0.03		2.15
	87.7 ± 19.2	12.3 ± 1.3	4.41 ± 0.59	0.76 ± 0.04	1.36
**2 to 3**	100		1.45 ± 0.05		2.31
	74.5 ± 10.2	25.5 ± 11.1	2.71 ± 0.60	0.94 ± 0.14	1.93
**3 to 2**	100		1.07 ± 0.04		0.39
	72.5 ± 12.0	27.5 ± 14.0	1.90 ± 0.45	0.72 ± 0.12	0.3

Supplementary Table 8: Mono-exponential and bi-exponential fitting results from the dwell time analysis of $A_{2A}AR$[R291Q] without ligand added (apo) and with ligands in the absence of mini-G_S.

States	A ± sd (%)		k ± sd (1/s)		Reduced χ^2
**A_{2A}AR[I92N]—APO**					
**1 to 2**	100		1.37 ± 0.06		1
	90.4 ± 10.6	9.6 ± 1.4	3.99 ± 0.41	0.58 ± 0.04	0.39
**2 to 1**	100		1.67 ± 0.07		1.85
	93.3 ± 14.1	6.7 ± 1.5	3.16 ± 0.35	0.59 ± 0.06	1.04
**2 to 3**	100		1.31 ± 0.07		0.96
	76.9 ± 23.4	23.1 ± 5.9	4.28 ± 1.43	0.93 ± 0.10	0.53
**3 to 2**	100		1.17 ± 0.06		1.07
	77.5 ± 53.1	22.5 ± 58.9	1.55 ± 0.73	0.78 ± 0.51	1.05
**A_{2A}AR[I92N]—NECA**					
**1 to 2**	100		1.75 ± 0.08		2.14
	93.2 ± 9.6	6.8 ± 1.2	3.47 ± 0.32	0.49 ± 0.04	0.81
**2 to 1**	100		0.94 ± 0.04		0.98
	96.0 ± 5.9	4.0 ± 3.8	1.12 ± 0.10	0.31 ± 0.13	0.87
**2 to 3**	100		1.37 ± 0.07		0.94
	74.0 ± 37.0	26.0 ± 5.3	5.77 ± 2.51	1.08 ± 0.10	0.56
**3 to 2**	100		0.93 ± 0.05		0.93
	79.6 ± 11.1	20.4 ± 9.3	1.82 ± 0.42	0.53 ± 0.09	0.44
**A_{2A}AR[I92N]—ZM241385**					
**1 to 2**	100		1.42 ± 0.06		1.45
	89.5 ± 11.8	10.5 ± 1.6	4.24 ± 0.50	0.67 ± 0.05	0.64
**2 to 1**	100		1.10 ± 0.04		1.35
	77.5 ± 8.5	22.5 ± 7.1	2.29 ± 0.41	0.67 ± 0.08	0.75
**2 to 3**	100		1.42 ± 0.07		0.5
	78.1 ± 21.9	21.9 ± 4.4	5.12 ± 1.40	1.00 ± 0.09	0.24
**3 to 2**	100		1.06 ± 0.05		1.07
	95.9 ± 7.1	4.1 ± 4.7	1.25 ± 0.14	0.33 ± 0.17	0.92

Supplementary Table 9: Mono-exponential and bi-exponential fitting results from the dwell time analysis of A_{2A}AR[I92N] without ligand added (apo) and with ligands in the absence of mini-G_S.

States	A ± sd (%)		k ± sd (1/s)		Reduced χ^2
$A_{2A}AR$[D52N]—APO					
1 to 2	100		0.86 ± 0.04		1.36
	93.3 ± 9.0	6.7 ± 1.2	1.67 ± 0.14	0.24 ± 0.02	0.42
2 to 1	100		1.26 ± 0.05		1.12
	85.2 ± 25.4	14.8 ± 2.1	5.00 ± 0.99	0.88 ± 0.06	0.58
2 to 3	100		1.55 ± 0.11		0.28
	93.4 ± 392	6.6 ± 1.8	9.58 ± 12.6	1.27 ± 0.15	0.15
3 to 2	100		1.84 ± 0.13		0.44
	91.5 ± 22.3	8.5 ± 4.8	3.39 ± 0.73	0.79 ± 0.20	0.29
$A_{2A}AR$[D52N]—NECA					
1 to 2	100		0.98 ± 0.05		3
	94.7 ± 14.4	5.3 ± 0.7	3.33 ± 0.33	0.35 ± 0.02	0.86
2 to 1	100		1.01 ± 0.04		1.03
	85.4 ± 10.6	14.6 ± 2.9	2.51 ± 0.33	0.55 ± 0.05	0.46
2 to 3	100		1.28 ± 0.09		1.52
	87.6 ± 27.7	12.4 ± 4.7	3.38 ± 0.92	0.69 ± 0.12	0.53
3 to 2	100		1.36 ± 0.09		0.87
	90.7 ± 16.9	9.3 ± 4.9	2.40 ± 0.48	0.54 ± 0.13	0.36
$A_{2A}AR$[D52N]—ZM241385					
1 to 2	100		0.80 ± 0.04		3.81
	92.5 ± 14.5	7.5 ± 1.0	2.81 ± 0.32	0.34 ± 0.02	0.68
2 to 1	100		1.12 ± 0.04		1
	86.8 ± 31.2	13.2 ± 1.6	5.51 ± 1.17	0.82 ± 0.05	0.53
2 to 3	100		1.41 ± 0.11		0.73
	96.1 ± 649	3.9 ± 1.1	10.1 ± 18.7	1.12 ± 0.14	0.46
3 to 2	100		1.71 ± 0.13		0.63
	91.3 ± 18.2	8.7 ± 9.0	2.61 ± 0.66	0.72 ± 0.31	0.49

Supplementary Table 10: Mono-exponential and bi-exponential fitting results from the dwell time analysis of $A_{2A}AR$[D52N] without ligand added (apo) and with ligands in the absence of mini-G_S.

States	A ± sd (%)		k ± sd (1/s)		Reduced χ^2
**A_{2A}AR—APO with Mini-G_S**					
**1 to 2**	100		1.19 ± 0.05		2.89
	93.6 ± 7.9	6.4 ± 0.7	2.97 ± 0.21	0.33 ± 0.02	0.93
**2 to 1**	100		0.78 ± 0.02		1.34
	84.3 ± 5.7	15.7 ± 4.6	1.32 ± 0.14	0.39 ± 0.04	0.72
**2 to 3**	100		1.20 ± 0.08		1.55
	80.9 ± 24.6	19.1 ± 4.9	4.11 ± 1.27	0.78 ± 0.09	0.73
**3 to 2**	100		1.39 ± 0.08		0.52
	83.3 ± 19.2	16.7 ± 23.2	2.01 ± 0.62	0.78 ± 0.35	0.42
**A_{2A}AR—NECA with Mini-G_S**					
**1 to 2**	100		2.45 ± 0.09		1.74
	95.0 ± 10.3	5.0 ± 0.5	5.40 ± 0.40	0.57 ± 0.03	0.66
**2 to 1**	100		0.86 ± 0.03		1.9
	84.9 ± 5.9	15.1 ± 5.3	1.37 ± 0.15	0.43 ± 0.05	1.43
**2 to 3**	100		1.35 ± 0.06		2.1
	84.9 ± 33.6	15.1 ± 1.9	7.56 ± 1.96	1.01 ± 0.06	1.26
**3 to 2**	100		0.85 ± 0.03		1.29
	87.7 ± 7.0	12.3 ± 4.2	1.43 ± 0.17	0.38 ± 0.05	0.5
**A_{2A}AR—ZM241385 with Mini-G_S**					
**1 to 2**	100		1.60 ± 0.05		2.09
	94.4 ± 6.4	5.6 ± 0.5	3.66 ± 0.20	0.39 ± 0.02	0.55
**2 to 1**	100		0.89 ± 0.02		1.28
	81.8 ± 5.0	18.2 ± 4.1	1.60 ± 0.15	0.48 ± 0.04	0.65
**2 to 3**	100		1.32 ± 0.06		1.16
	78.6 ± 27.4	21.4 ± 3.3	6.13 ± 1.77	1.00 ± 0.07	0.59
**3 to 2**	100		1.18 ± 0.05		0.54
	91.5 ± 9.5	8.5 ± 10.4	1.51 ± 0.22	0.55 ± 0.23	0.44

Supplementary Table 11: Mono-exponential and bi-exponential fitting results from the dwell time analysis of A_{2A}AR without ligand added (apo) and with ligands in the presence of mini-G_S.

States	A ± sd (%)		k ± sd (1/s)		Reduced χ^2
**A_{2A}AR[R291Q]—APO with Mini-G_S**					
**1 to 2**	100		1.47 ± 0.05		1.48
	91.5 ± 5.9	8.5 ± 1.8	2.44 ± 0.18	0.52 ± 0.05	0.56
**2 to 1**	100		1.24 ± 0.04		0.75
	85.7 ± 10.6	14.3 ± 4.5	2.38 ± 0.34	0.69 ± 0.08	0.47
**2 to 3**	100		1.55 ± 0.06		1.07
	63.7 ± 13.0	36.3 ± 15.7	3.23 ± 1.19	1.16 ± 0.16	0.92
**3 to 2**	100		1.06 ± 0.04		1.23
	90.4 ± 7.5	9.6 ± 6.9	1.43 ± 0.19	0.45 ± 0.12	0.96
**A_{2A}AR[R291Q]—NECA with Mini-G_S**					
**1 to 2**	100		1.32 ± 0.05		1.22
	89.0 ± 19.8	11.0 ± 1.2	6.77 ± 1.03	0.85 ± 0.05	0.59
**2 to 1**	100		1.00 ± 0.03		0.96
	84.8 ± 6.3	15.2 ± 3.8	1.88 ± 0.21	0.49 ± 0.05	0.49
**2 to 3**	100		1.26 ± 0.06		0.8
	82.3 ± 40.3	17.7 ± 2.6	7.14 ± 2.43	0.97 ± 0.07	0.48
**3 to 2**	100		1.16 ± 0.05		2.11
	98.9 ± 6.5	1.1 ± 1.0	1.29 ± 0.08	0.13 ± 0.12	1.79
**A_{2A}AR[R291Q]—ZM241385 with Mini-G_S**					
**1 to 2**	100		1.93 ± 0.07		1.42
	92.8 ± 7.5	7.2 ± 1.4	3.22 ± 0.26	0.56 ± 0.05	0.39
**2 to 1**	100		0.87 ± 0.03		1.16
	92.0 ± 5.7	8.0 ± 4.0	1.17 ± 0.11	0.33 ± 0.07	0.77
**2 to 3**	100		1.28 ± 0.06		0.64
	69.6 ± 15.4	30.4 ± 9.5	3.36 ± 1.12	0.93 ± 0.11	0.45
**3 to 2**	100		1.06 ± 0.05		1.22
	96.4 ± 7.1	3.6 ± 3.6	1.26 ± 0.12	0.30 ± 0.15	1.01

Supplementary Table 12: Mono-exponential and bi-exponential fitting results from the dwell time analysis of A_{2A}AR[R291Q] without ligand added (apo) and with ligands in the presence of mini-G_S.

States	A ± sd (%)		k ± sd (1/s)		Reduced χ^2
**A_{2A}AR[I92N]—APO with Mini-G_S**					
**1 to 2**	100		1.37 ± 0.06		1.93
	93.5 ± 7.7	6.5 ± 1.2	2.46 ± 0.20	0.36 ± 0.04	0.5
**2 to 1**	100		0.98 ± 0.03		0.84
	99.0 ± 5.1	1.0 ± 1.0	1.06 ± 0.06	0.18 ± 0.11	0.74
**2 to 3**	100		1.17 ± 0.06		0.44
	81.1 ± 12.0	18.9 ± 7.8	2.41 ± 0.53	0.66 ± 0.11	0.2
**3 to 2**	100		1.06 ± 0.05		1.36
	97.4 ± 7.7	2.6 ± 4.1	1.18 ± 0.13	0.29 ± 0.23	1.32
**A_{2A}AR[I92N]—NECA with Mini-G_S**					
**1 to 2**	100		1.43 ± 0.06		2.3
	91.6 ± 16.0	8.4 ± 0.9	6.25 ± 0.74	0.73 ± 0.04	1.01
**2 to 1**	100		0.99 ± 0.03		0.58
	83.3 ± 6.8	16.7 ± 6.4	1.61 ± 0.21	0.52 ± 0.07	0.31
**2 to 3**	100		1.25 ± 0.06		1.74
	74.6 ± 25.9	25.4 ± 4.1	5.29 ± 1.68	0.97 ± 0.07	1.23
**3 to 2**	100		0.95 ± 0.04		0.8
	98.8 ± 5.9	1.2 ± 1.5	1.02 ± 0.07	0.19 ± 0.14	0.76
**A_{2A}AR[I92N]—ZM241385 with Mini-G_S**					
**1 to 2**	100		1.27 ± 0.05		1.91
	86.7 ± 10.2	13.3 ± 1.8	3.73 ± 0.43	0.64 ± 0.04	0.85
**2 to 1**	100		1.10 ± 0.04		0.5
	81.4 ± 11.6	18.6 ± 5.8	2.27 ± 0.40	0.68 ± 0.08	0.26
**2 to 3**	100		1.38 ± 0.05		0.84
	97.2 ± 6.3	2.8 ± 5.1	1.52 ± 0.15	0.46 ± 0.35	0.77
**3 to 2**	100		1.18 ± 0.05		0.71
	95.8 ± 6.5	4.2 ± 4.6	1.39 ± 0.14	0.40 ± 0.18	0.55

Supplementary Table 13: Mono-exponential and bi-exponential fitting results from the dwell time analysis of A_{2A}AR[I92N] without ligand added (apo) and with ligands in the presence of mini-G_S.

States	A ± sd (%)		k ± sd (1/s)		Reduced χ^2
**A_{2A}AR[D52N]—APO with Mini-G_S**					
**1 to 2**	100		0.85 ± 0.04		1.55
	91.9 ± 9.8	8.1 ± 1.3	1.84 ± 0.18	0.28 ± 0.02	0.54
**2 to 1**	100		1.33 ± 0.05		0.86
	89.8 ± 17.0	10.2 ± 1.5	4.29 ± 0.56	0.72 ± 0.05	0.31
**2 to 3**	100		1.64 ± 0.13		0.58
	94.4 ± 128	5.6 ± 1.5	8.31 ± 4.31	1.14 ± 0.14	0.18
**3 to 2**	100		1.73 ± 0.11		0.64
	90.5 ± 224	9.5 ± 2.4	9.62 ± 8.47	1.43 ± 0.16	0.49
**A_{2A}AR[D52N]—NECA with Mini-G_S**					
**1 to 2**	100		1.05 ± 0.05		1.84
	93.8 ± 12.6	6.2 ± 0.9	2.35 ± 0.24	0.29 ± 0.02	0.52
**2 to 1**	100		1.05 ± 0.04		1.14
	84.7 ± 12.7	15.3 ± 3.1	2.76 ± 0.42	0.60 ± 0.05	0.51
**2 to 3**	100		1.86 ± 0.14		1.17
	91.8 ± 19.8	8.2 ± 7.4	2.92 ± 0.69	0.78 ± 0.29	0.87
**3 to 2**	100		1.64 ± 0.12		0.8
	90.5 ± 17.7	9.5 ± 7.7	2.63 ± 0.62	0.71 ± 0.24	0.44
**A_{2A}AR[D52N]—ZM241385 with Mini-G_S**					
**1 to 2**	100		0.84 ± 0.04		1.98
	91.8 ± 19.8	8.2 ± 1.5	2.79 ± 0.44	0.39 ± 0.04	0.91
**2 to 1**	100		1.17 ± 0.05		0.97
	91.2 ± 9.2	8.8 ± 3.0	2.02 ± 0.23	0.47 ± 0.07	0.54
**2 to 3**	100		1.45 ± 0.11		0.56
	94.4 ± 14.9	5.6 ± 7.7	1.91 ± 0.40	0.52 ± 0.31	0.4
**3 to 2**	100		1.81 ± 0.14		1.07
	92.1 ± 20.9	7.9 ± 4.8	3.03 ± 0.66	0.67 ± 0.18	0.62

Supplementary Table 14: Mono-exponential and bi-exponential fitting results from the dwell time analysis of A_{2A}AR[D52N] without ligand added (apo) and with ligands in the presence of mini-G_S.

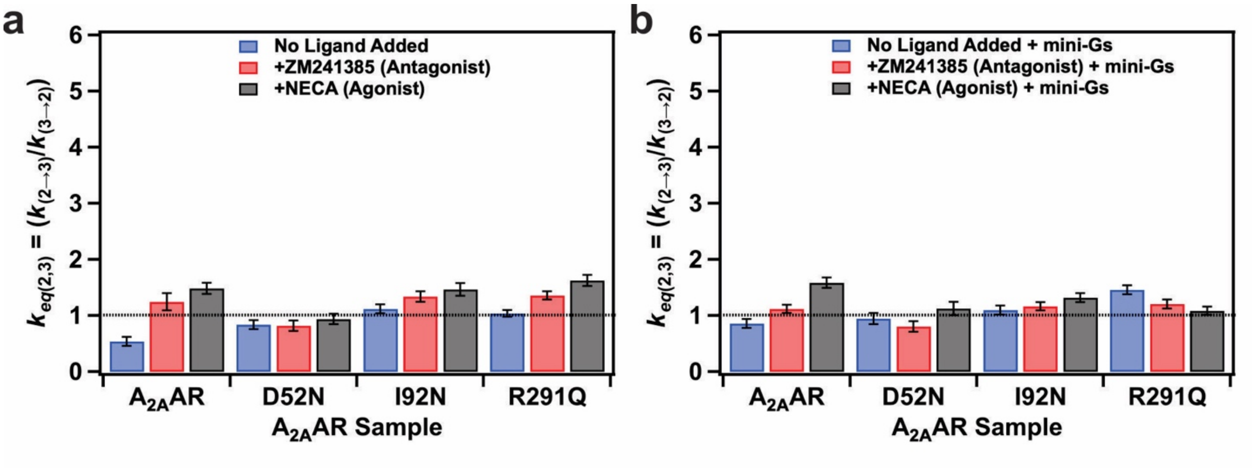

Supplementary Fig. 7. An expanded view of Fig. 7 panels c and d, here shown with the same Y axis range as Fig. 7 panels a and b. Error bars indicate the standard deviation.
