# Supplementary material for: Single-molecule visualization of human A2A adenosine receptor activation by a G protein and constitutively activating mutations
Source: Commun Biol. 2023 Nov 30;6:1218. doi: 10.1038/s42003-023-05603-6 (PMC10689853; doi:10.1038/s42003-023-05603-6)
Supplement: Supplementary file 2 — Description of Supplementary Materials [file 42003_2023_5603_MOESM2_ESM.docx]

**Description of Additional Supplementary Files**

**File name:** Supplementary Data 1

**Description:** The data used to draw the figures in the main text and supplementary materials.
